# Pharmacovigilance profiles of three generations of mineralocorticoid receptor antagonists and network toxicology analysis

**DOI:** 10.3389/fmed.2026.1797331

**Published:** 2026-06-23

**Authors:** Hai-Hong Lin, Wen-Hui Cui, Feng Xu, Jia-Yi He, Liu-Cheng Li, Jun-Jie Xu, Xin Jiang, Kai-Li Mao

**Affiliations:** 1 Department of Pharmacy, Wenling TCM Hospital Affiliated to Zhejiang Chinese Medical University, Wenling, China; 2 Department of Pharmacy, Sinopharm Tongmei General Hospital, Datong, China; 3 Department of Pharmacy, Ningbo No 2 Hospital, Wenzhou Medical University, Ningbo, China; 4 Department of Pharmacy, The First People's Hospital of Wuyi County, Jinhua, China; 5 Department of Pharmacy, Sir Run Run Shaw Hospital, Zhejiang University School of Medicine, Hangzhou, China; 6 Department of Pharmacy, The Quzhou Affiliated Hospital of Wenzhou Medical University, Quzhou People’s Hospital, Quzhou, China

**Keywords:** adverse drug reaction, FAERS database, mineralocorticoid receptor antagonists, pharmacovigilance, real-world evidence

## Abstract

**Introduction:**

Mineralocorticoid receptor antagonists, spironolactone, eplerenone, and finerenone, are cornerstone therapies in cardiovascular and renal diseases. While their efficacy is established, a systematic, head-to-head comparison of their post-marketing safety profiles based on large-scale real-world data is lacking.

**Methods:**

This study comprehensively compared the post-marketing safety profiles of spironolactone, eplerenone, and finerenone using 11,556 primary suspect reports from the FAERS database (Q1 2004–Q3 2025).

**Results:**

Disproportionality analyses revealed distinct adverse event (AE) signatures. Spironolactone exhibited the broadest safety profile, with disproportionate reporting indicating congenital malformations, acute pulmonary edema, serious cutaneous reactions, and antiandrogenic effects including rare signals such as endometriosis in males. Eplerenone showed a more focused yet severe profile, with signals for serious cardiac events and renal/hepatic failure. Finerenone demonstrated the most targeted profile, with signals predominantly confined to renal and metabolic domains. Hyperkalemia was a consistent moderate-priority signal across all three drugs, and several high-signal, unlabeled AEs were identified. Network toxicology analysis further supported these findings by linking shared and drug-specific molecular targets to key pathways (e.g., aldosterone-regulated sodium reabsorption, MAPK signaling).

**Discussion:**

These hypothesis-generating findings underscore the need for personalized safety monitoring, potential label updates, and independent validation in other pharmacovigilance databases before clinical or regulatory conclusions can be drawn.

## 1 Introduction

At present, there are three mineralocorticoid receptor antagonists (MRA) that are widely used in clinical practice: spironolactone, eplerenone, and finerenone. Spironolactone was the first MRA to be developed, and it is widely used to treat hypertension, primary hyperaldosteronism, and heart failure. It is also used to treat other conditions related to aldosteronism (1). Spironolactone, a synthetic 17-lactone, has been in clinical use as a non-selective MRA since its introduction in 1960. It has remained relevant for over six decades (2, 3).

Spironolactone is used to treat hypertension and acne in women due to its antagonistic effects on progesterone and androgen receptors (4, 5). Eplerenone, an aldosterone antagonist, increases survival in stable patients with symptomatic heart failure and reduced ejection fraction following an acute myocardial infarction. It was approved by the FDA in 2002. Eplerenone has also been shown to effectively reduce blood pressure and minimize the risk of fatal and nonfatal cardiovascular events, especially myocardial infarction and stroke (6). Both spironolactone and aldosterone bind to the mineralocorticoid receptor, causing sodium and fluid retention. Eplerenone acts similarly. Compared to aldosterone, eplerenone has a shorter half-life and can be metabolized into inactive byproducts that are easier to flush out of the body. In clinical practice, spironolactone’s sexual side effects are the primary reason hypertensive patients struggle to adhere to their medication, but eplerenone can significantly reduce the incidence of such adverse events (AEs). Earlier clinical studies and a randomized controlled trial revealed that the most common AEs associated with eplerenone treatment for heart failure with reduced ejection fraction (HFrEF) are hyperkalemia and elevated creatinine levels. These AEs are listed in the eplerenone prescribing information (7). Finerenone, a new non-steroidal MRA (NS-MRA), which was approved in the United States in 2021, has a clear renoprotective effect on diabetic patients with chronic kidney disease (CKD). The long-term use of finerenone can significantly reduce the urinary albumin/creatinine ratio. Compared with traditional steroid MRAs, it has little effect on serum potassium (8). Spironolactone and eplerenone are traditional steroid MRA. However, they have not been extensively studied in the field of chronic kidney disease (CKD) due to the risk of sex steroid receptor cross-reactivity and hyperkalemia (9–11). The adverse effects observed in clinical trials may not accurately reflect the actual results, as trials are conducted under widely varying conditions. Therefore, careful monitoring of AEs and the collection of real-world data are essential for creating valid drug reference standards and ensuring that drugs are used optimally to maximize patient benefits and minimize risks.

The U. S. Food and Drug Administration’s AE Reporting System (FAERS) is a publicly accessible database designed to help the FDA monitor the safety of drugs and therapeutic biological products after they have been marketed (12). This database enables the quantitative evaluation of reports via signal detection. A ‘signal’ indicates a drug-related AE and is analyzed to determine whether it is an AE. Researchers use a variety of particle-based analysis algorithms, such as the Reporting Odds Ratio (ROR), Proportional Reporting Ratio (PRR), Bayesian Confidence Propagation Neural Network (BCPNN), and Empirical Bayesian Geometric Mean (EBGM), to evaluate the risk of AEs and detect the strength of the signal of AEs associated with medical products. The ROR, PRR, BCPNN, and EBGM algorithms are commonly used in non-linear analyses and are currently in widespread use by the Health Products Regulatory Agency, the Netherlands PharmacoVigilance Center, This allows researchers to analyze a wide range of drugs and diseases, identify potential AEs of particular concern, and provide guidance on clinical medication and treatment strategies. The World Health Organization, and the FDA (13).

In this study, we analyzed AEs related to spironolactone, eplerenone and finerenone, reported from Q1 2004 to Q3 2025, using data mined from the FAERS database. We used the ROR and PRR algorithms to analyze the relationship between drugs and AEs, thereby identifying AEs. Next, we used the BCPNN algorithm to construct a joint probability model linking the drugs to the AEs identified through ROR and PRR. Finally, we used the EBGM algorithm to convert these risk associations into risk indices, identifying significant, high-risk combinations of drugs and AEs as recognized by the aforementioned algorithms. Additionally, we conducted a comparative analysis of the AEs associated with the two drugs, identifying potential new AE signals for further investigation. It is important to note that, as disproportionality analysis in spontaneous reporting systems detects reporting imbalances rather than causal relationships, all findings reported here should be interpreted as hypothesis-generating. The aim of this research is to generate signals that can inform future confirmatory studies and promote the rational clinical use of the drug in question. However, the results should not be overinterpreted as definitive comparative safety assessments.

## 2 Materials and methods

### 2.1 Data source

The FAERS database has been openly available since 2004 and is updated every 3 months. It compiles data on post-market AEs. The seven separate files that make up the FAERS dataset comprise patient demographic and administrative details (DEMO); drug-specific information (DRUG); coded representations of reported AEs (REAC); patient outcome information (OUTC); sources of reports (RPSR); therapy initiation and cessation dates for reported drugs (THER); and indications for drug administration (INDI). In this study, we conducted a retrospective pharmacovigilance analysis using FAERS data from the first quarter (Q1) of 2004 to the third quarter (Q3) of 2025. Spironolactone, eplerenone, and finerenone were used as keywords in a thorough search. We also extracted patient demographic and clinical details, such as sex, age, reporting region, reporter type, report date, and AE outcomes associated with spironolactone, eplerenone, and finerenone. In accordance with the Medical Dictionary for Regulatory Activities (MedDRA) (version 25.1), AEs were classified using preferred terms (PTs) from the FAERS database. Each PT can be linked to multiple High-Level Terms, High-Level Group Terms and System Organ Classes (SOCs) to ensure the correct PT names are used in the FAERS database (Supplementary Figure 1).

### 2.2 Data processing

Within the FAERS database, a single AE case may be reported multiple times, resulting in several entries for the same patient. Therefore, data cleaning procedures were implemented prior to analysis. The following measures were taken to ensure the completeness and accuracy of the data. Data cleaning and standardization: We used the Medex_UIMA_1.3.8 software to standardize and unify drug names in the database. This process consolidates data entries and reduces discrepancies. Duplicate removal: A two-step deduplication process was implemented. First, the data were normalized and cleaned to remove duplicate rows. Subsequently, deduplication was performed based on the latest FDA_DT if the CASEID and FDA_DT were identical. Specifically, duplicate entries were identified and removed using unique identifiers in FAERS, such as the CASEID (which identifies each case uniquely) and the ISR (Individual Safety Report) number (which tracks updates to the same case). For example, if multiple reports shared the same CASEID but had different ISR numbers (indicating updates or revisions), only the report with the latest submission date (i.e., the latest ISR version) was retained. This process ensured that each case was represented by its most up-to-date clinical information, eliminating redundant data from initial reports and subsequent amendments. It is important to note that drug names in FAERS are typically reported in free-text format, which may include both generic and brand names, as well as research codes. There is potential for spelling inconsistencies in this format. To address this issue, a comprehensive drug name reference was applied, encompassing all known generic and brand names, as well as study codes, for FDA-approved spironolactone, eplerenone and finerenone. Data validation: We cross-referenced the extracted data with the original reports to ensure accuracy. This process included verifying the consistency of drug names, AE terms, and patient demographic information. For reports with missing data (e.g., incomplete patient information), we only included those in which the MRA was clearly identified as the primary suspect (PS). Reports with missing preferred terms (PTs) or without a clearly designated primary suspect were excluded. Furthermore, reports with significant missing data that could affect the analysis were also excluded. These measures ensured the integrity and accuracy of the data extracted from the FAERS database, establishing a solid foundation for subsequent analysis. Data were extracted based on the names of the three MRAs: spironolactone, eplerenone, and finerenone.

### 2.3 Disproportionality analysis

Disproportionality analysis is a cornerstone technique of pharmacovigilance in the FAERS database. It evaluates the association between a drug and an AEs by comparing the disproportionality ratio of the target drug and AEs to that of other drug-AEs pairs. The disproportionality was quantified using a 2 × 2 contingency table (Supplementary Table 1) to detect potential pharmacovigilance risk signals. In this study, we used the frequency-based Reporting Odds Ratio (ROR) and Proportional Reporting Ratio (PRR) methods, as well as the Bayesian Confidence Propagation Neural Network (BCPNN) and Empirical Bayes Geometric Mean (EBGM) methods, to detect AE signals. The detailed formulas and thresholds of the four algorithms are presented in Supplementary Table 2. ROR signals are considered positive if the lower limit of the 95% CI is greater than 1 and a is greater than or equal to 3 (the number of reports containing both the suspect drug and the suspect AE). A PRR ≥ 2, a *χ*^2^ ≥ 4, and an a ≥ 3 are considered positive signals. EBGM05 > 2 is also considered a positive signal. A positive BCPNN signal is indicated by IC025 > 0; a higher IC025 value suggests a stronger signal and a higher correlation between the target drug and the AE. In this study, a signal was confirmed only when it was detected by all four algorithms.

This study adheres to standard pharmacovigilance reporting practices for disproportionality analysis using spontaneous reporting databases. No single reporting guideline formally covers retrospective FAERS-based disproportionality studies; therefore, the study follows conventional methodological standards consistent with recommendations from the European Medicines Agency’s Pharmacovigilance Risk Assessment Committee and the WHO Uppsala Monitoring Centre. The research team employed a rigorous methodology. This methodology ensured the findings were of high quality, reliable, and reproducible. It is important to note that a disproportionality analysis identifies statistical associations, rather than causal relationships. Throughout this manuscript, therefore, we use cautious language (e.g., ‘disproportionate reporting suggests,’ ‘signal indicates,’ ‘statistical association’) rather than definitive causal statements (e.g., ‘causes,’ ‘increases risk,’ ‘leads to’). Comparisons of signal strength between drugs should not be misinterpreted as definitive rankings of comparative toxicity.

### 2.4 Classification and prioritization of relevant disproportionality signals

AEs that showed a significant association in all four disproportionality analyses (ROR, PRR, BCPNN, and EBGM) were ranked based on a semiquantitative evaluation using the criteria in Supplementary Table 3. Each criterion was assigned a score of 0, 1, or 2 points, resulting in a total possible score ranging from 0 to 8 points for each AE. Scores of 0–2, 3–5, and 6–8 indicated AEs of low, moderate, or high priority, respectively. The criteria were selected to reflect multiple dimensions of clinical and methodological relevance. Each criterion contributes independently to the overall priority score (14). The criteria and their respective scoring thresholds were as follows:

1. Clinical relevance: This was assessed using the European Medicines Agency’s lists of important medical events (IMEs), which denote serious events, and designated medical events (DMEs), which represent rare but serious events with a high likelihood of being drug-related (15). AEs classified as DMEs received two points, IMEs received one point, and other events received zero points. This criterion prioritizes AEs based on their potential clinical severity and established drug association.
2. Reporting rate (calculated as cases/non-cases): It was assessed by comparing the proportion of the AE of interest to that of other AEs (i.e., the ratio of cases to non-cases) (16). A score of 2 was assigned for a rate >10%, 1 point for a rate of 1–10%, and 0 points for a rate of 0–1%. Higher scores suggest a greater reporting burden, which could improve the reliability of the signal.
3. Signal stability: The stability of disproportionate signals is evaluated based on their consistency and reliability across various analyses. The consistency of the results across four different disproportionality analyses was assessed. Two points were given if the signal was present in all four analyses, one point if it was present in three, and zero points if it was present in only two or fewer (17). This criterion guarantees that prioritized signals remain robust when using multiple statistical approaches.
4. Reported case fatality rate: This metric measures the proportion of reports in which death was recorded compared to all AEs. The proportion of reports with death as the outcome was evaluated. Reports with a rate greater than 50% received two points, those with a rate between 25 and 50% received one point, and those with a rate less than 25% received zero points (18). This metric is used as an indicator of the severity and potential clinical impact of the AE.

The total score for each AE was calculated by summing the points from the four criteria. This scoring framework was adapted from established pharmacovigilance approaches that use multicriteria semi-quantitative assessments to prioritize signals. Similar methodologies have previously been used to integrate clinical relevance, statistical robustness and public health impact into a unified ranking system, thereby supporting systematic and transparent decision-making in signal management. The prioritized AEs were then reviewed to inform further clinical evaluation.

### 2.5 Ethics and study approval

This study used data from the FAERS database, which is a publicly available, anonymized resource designed to support the FDA-approved drug safety surveillance program. The database is accessible to the public for research purposes. Because the data is anonymized and intended for surveillance and research, Institutional Review Board approval and patient consent were not required.

### 2.6 Network toxicology analysis

This network toxicology analysis was performed to generate mechanistic hypotheses for the AE signals identified in the disproportionality analysis. This provides *in silico* evidence to inform future experimental validation.

#### 2.6.1 Target collection

Using “spironolactone,” “eplerenone,” and finerenone as keywords, the toxicogenomics database CTD^1^, STITCH^2^, Super-PRED^3^, SEA^4^ and PharmMap^5^ Search drug targets. Retrieve disease targets for the keywords “acute kidney injury” from the OMIM^6^, GeneCards^7^ and TTD^8^ databases, integrate all targets of drugs and diseases, and obtain the drug-disease associated target dataset.

#### 2.6.2 PPI network construction

The drug and disease-associated target dataset was imported into the STRING platform^9^ for PPI analysis. The interaction network results provided in TSV format were imported into Cytoscape software (version 3.9.0) for visualizing target protein interactions and calculating various network properties, such as degree, betweenness centrality, etc. The selection criteria are as follows: degree ≥ twice the median, betweenness centrality (Betweenness Centrality) ≥ median. A PPI regression curve was plotted with betweenness centrality as the *y*-axis and degree as the *x*-axis.

#### 2.6.3 GO and KEGG enrichment analysis

The drug and disease associated target dataset was imported into the DAVID platform^10^, for gene ontology (GO) functional and Kyoto Encyclopedia of Genes and Genomes (KEGG) pathway enrichment analysis. The analysis data on biological processes (BP), cellular components (CC), molecular functions (MF), and pathways were selected based on *p*-values from low to high to identify the top 10 terms, generating GO bar charts and KEGG bar charts.

### 2.7 Statistical analysis

Disproportionality analysis is a key pharmacovigilance technique for identifying signals of spontaneous AEs. In this study, we investigated potential correlations between spironolactone, eplerenone, finerenone, and their respective AEs using frequentist and Bayesian methods, as well as established statistical tools such as the ROR, PRR, BCPNN, and EBGM. Our analysis was limited to AE signals with a minimum of three records that met all four algorithmic criteria simultaneously, notably that the lower limit of the 95% confidence interval (CI) was greater than 1.0. To minimize the likelihood of false positives arising from infrequent events with small sample sizes, we applied a statistical shrinkage technique. At the PT level, we categorized the number and types of AEs by organ toxicity. Then, the *Z*-test and Fisher’s exact test for proportions were used to compare the proportions of AEs and AE types between spironolactone, eplerenone, and finerenone. All data processing and statistical analyses were carried out using R software (version 4.3.3).

## 3 Results

### 3.1 Clinical characteristics of the reports

**Figure 1:**
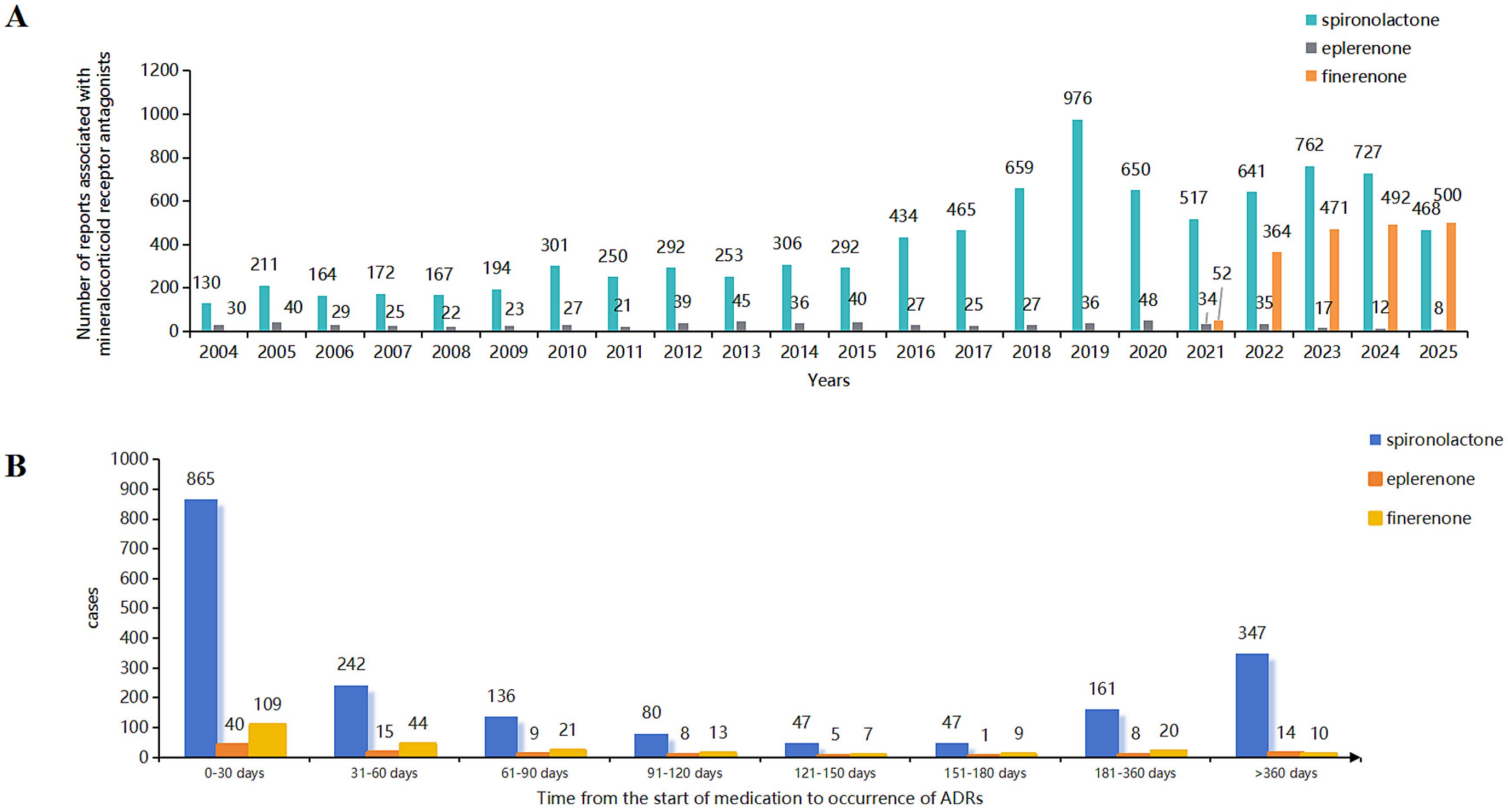
Number of reports associated with mineralocorticoid receptor antagonists (spironolactone, eplerenone and finerenone) by year **(A)**, and time-to-onset distribution of AEs **(B)**, based on FAERS data from Q1 2004 to Q3 2025. **(A)** Annual report counts show temporal trends in reporting frequency. **(B)** Time-to-onset was calculated as the interval (in days) between the start date of drug administration and the date of event onset, and was categorized into predefined intervals (≤30, 31–60, 61–90, 91–120, 121–150, 151–180, 181–270, 271–360, and >360 days). AEs, adverse events; FAERS, FDA Adverse Event Reporting System.

**Figure 2:**
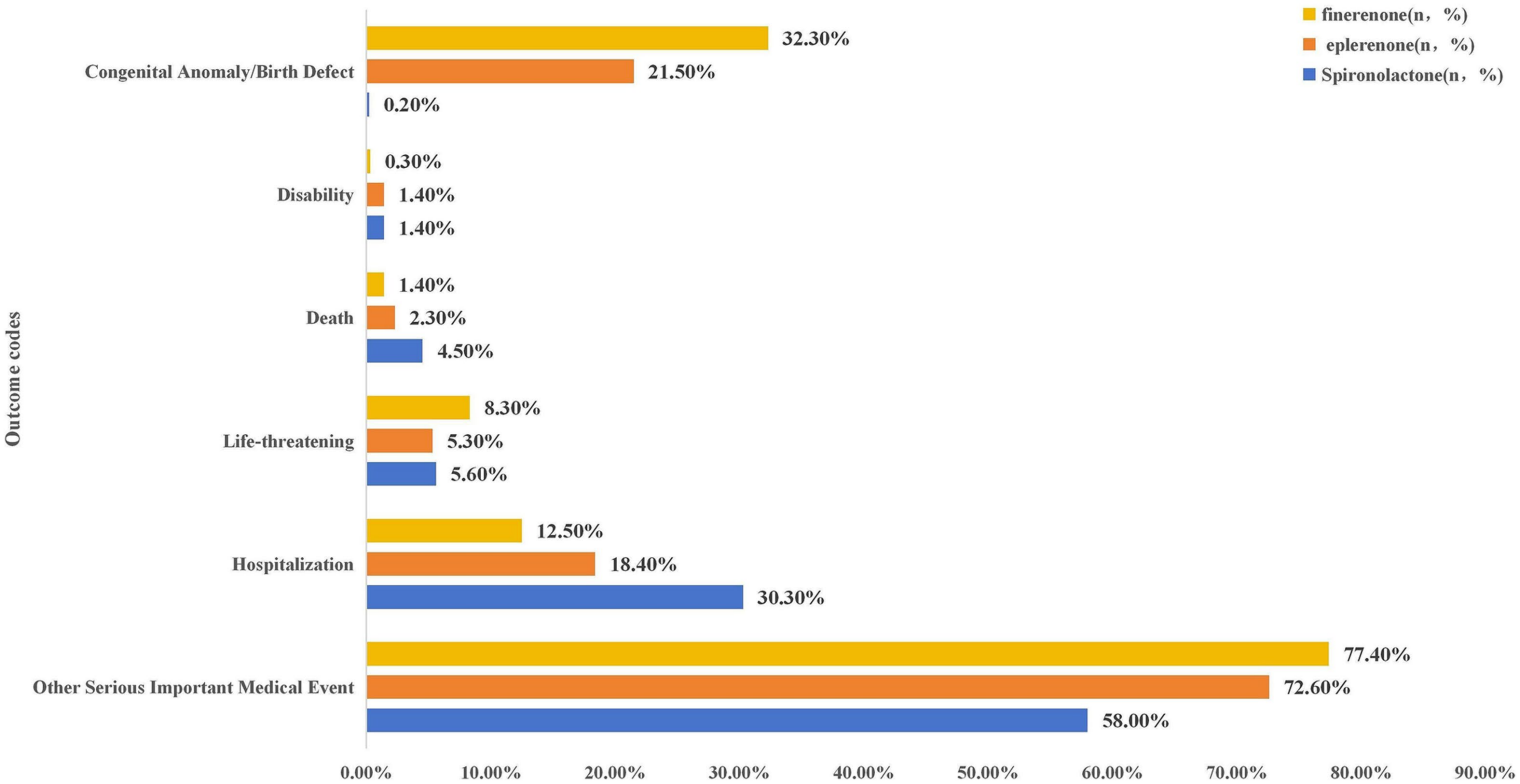
Clinical outcomes reported for spironolactone, eplerenone and finerenone from the FAERS database (Q1 2004 to Q3 2025). The outcome categories are defined according to the FAERS coding system and include: other serious and important medical events (including life-threatening events, hospitalization, disability, congenital anomalies and interventions required); hospitalization (initial or prolonged); life-threatening events; death; disability; and congenital anomalies/birth defects. Percentages represent the proportion of each outcome within the total number of reports for each drug. FAERS, FDA Adverse Event Reporting System.

This study comprehensively analyzed AE reports associated with three MRA from the FAERS database spanning from 2004 to 2025. The data revealed several key demographic and clinical characteristics among the 11,556 primary suspect reports (Table 1). The number of annual reports fluctuated. The highest points were 976 cases of spironolactone in 2019, 48 cases of eplerenone in 2020, and 500 cases of finerenone in 2025. The lowest points were 130 cases of spironolactone in 2004, eight cases of eplerenone in 2025, and 52 cases of finerenone in 2021 (Figure 1A). Among all AEs, a higher percentage of female patients (49.8%) than male patients (41.4%) were reported to have used spironolactone. In contrast, eplerenone showed the opposite trend, with a higher percentage of males (66.6%) than females (21.5%). Additionally, more male patients (47.7%) than female patients (32.3%) were reported to have used finerenone. The largest percentage of reports for spironolactone (40.6%), eplerenone (39%), and finerenone (33.2%) occurred in patients aged 65–85. A comparison of reported outcomes among the three medications revealed notable differences. For spironolactone, 58% of reports were categorized as “other serious important medical events,” while fatal outcomes accounted for 4.5%. In contrast, eplerenone was associated with “other serious important medical events” in 72.6% of reports, with death occurring in 2.3% of cases. Finerenone showed the highest proportion of reports classified as “other serious important medical events” at 77.4%, and the lowest fatality rate at 1.4% (Figure 2).

**Table 1:** The characteristics of reports associated with Spironolactone, eplerenone and finerenone from the FAERS database (2004 Q1 to 2025 Q3).

Items	Spironolactone (*n*, %)	Eplerenone (*n*, %)	Finerenone (*n*, %)
Total	9,031	646	1,879
Sex			
Female	4,493 (49.8%)	139 (21.5%)	607 (32.3%)
Male	3,741 (41.4%)	430 (66.6%)	897 (47.7%)
Missing	797 (8.8%)	77 (11.9%)	375 (20%)
Weight			
<50 kg	244 (2.7%)	3 (0.5%)	5 (0.3%)
50–100 kg	2,301 (25.5%)	166 (25.7%)	122 (6.5%)
>100 kg	467 (5.2%)	39 (6.0%)	10 (0.5%)
Missing	6,019 (66.6%)	438 (67.8%)	1742 (92.7%)
Age			
<18	170 (1.9%)	2 (0.3%)	39 (2.1%)
18–64	2,459 (27.2%)	148 (22.9%)	243 (12.9%)
65–85	3,671 (40.6%)	252 (39.0%)	623 (33.2%)
>85	886 (9.8%)	28 (4.3%)	50 (2.7%)
Missing	1845 (20.4%)	216 (33.4%)	924(49.2%)
Reporters			
Consumer	2,164 (24.0%)	184 (28.5%)	517 (27.5%)
Health-Professional	714 (7.9%)	32 (5.0%)	586(31.2%)
Pharmacist	1810 (20.0%)	215 (33.3%)	705(37.5%)
Physician	2,753 (30.5%)	83 (12.8%)	70 (3.7%)
Missing	1,590 (17.6%)	132 (20.4%)	1(0.1%)
Country of the reporter (top 5)			
US (United States)	3,171(35.1%)	212 (32.8%)	1,285 (68.4%)
FR (France)	1792 (19.8%)	135 (20.9%)	0
UK (United Kingdom)	873 (9.7%)	43 (6.7%)	0
JP (JAPAN)	533 (5.9%)	0	169 (9.0%)
DE (GERMANY)	385(4.3%)	78 (12.1%)	0
Outcome codes			
Other Serious Important Medical Event	5,242 (58.0%)	469 (72.6%)	1,455 (77.4%)
Hospitalization	2,735 (30.3%)	119 (18.4%)	235 (12.5%)
Life-threatening	509 (5.6%)	34 (5.3%)	156 (8.3%)
Death	402 (4.5%)	15 (2.3%)	27 (1.4%)
Disability	127 (1.4%)	9 (1.4%)	6(0.3%)
Congenital Anomaly/Birth Defect	16 (0.2%)	0	0

Data are presented as *n* (%).

FAERS, FDA Adverse Event Reporting System.

Pharmacists were the primary reporters for eplerenone and finerenone, accounting for 33.3 and 37.5% of the cases, respectively. Physicians were the primary reporters of spironolactone cases, contributing 30.5% of them. Geographically, The United States was the main reporting country for spironolactone (35.1%), finerenone (68.4%), and eplerenone (32.8%). Finerenone had the highest concentration. The time frame for AE occurrence varied greatly (Figure 1B). The highest case frequency for spironolactone was observed in the 0–30 day interval (*n* = 865), followed by the >360 day interval (*n* = 347). Case counts were notably lower across intermediate time periods, with the lowest count (47 cases) noted in the 121–180 day interval. Regarding eplerenone, the peak incidence was also in the 0–30 day interval (*n* = 40), followed by 15 cases in the 31–60 day interval. The lowest count was in the 151–180 day interval with one case reported. Similarly, for finerenone, case numbers peaked in the 0–30 day interval (*n* = 109), followed by 44 cases in the 31–60 day interval. The lowest frequency was observed in the 121–150 day interval with seven cases. In addition to the overall time-to-onset distribution, we conducted a stratified analysis of three high-priority signals in order to approximate exposure-response temporal patterns. Among spironolactone reports, hyperkalemia (*n* = 1,204) was distributed across all time intervals (≤30 days: 31.6%; 31–90 days: 34.1%; >90 days: 34.4%), whereas gynaecomastia (*n* = 255) predominantly manifested after prolonged exposure (>90 days: 58.8%), with a median onset of 215 days. For eplerenone, acute kidney injury (*n* = 49) occurred most frequently within the first 30 days (61.2%). For finerenone, acute kidney injury (*n* = 53) also exhibited an early-onset pattern (71.7% within ≤30 days), suggesting a potential acute haemodynamic effect rather than cumulative toxicity.

### 3.2 Disproportionality analysis at the SOC level

The signal strengths of reports of spironolactone, eplerenone, and finerenone at the SOC level are outlined separately in Supplementary Table 4. The data analysis revealed that spironolactone-associated AEs affected 27 organ systems, highlighting the relatively common occurrence of spironolactone-related AEs. eplerenone-associated AEs also affected 27 organ systems. Three organ systems were affected by finerenone-associated AEs (Supplementary Table 4). These reported AEs spanned a wide range of System Organ Classes. The largest number of AEs were predominantly observed in general disorders and administration site conditions in both spironolactone (*n* = 3,255, EBGM05 = 0.71) and eplerenone (*n* = 217, EBGM05 = 0.66), Investigations involving finerenone had the highest number of AEs (*n* = 770, EBGM05 = 4.15) which is consistent with the known pharmacology of the drugs. Large numbers of AEs were also observed in metabolism and nutrition disorders (*n* = 2,979, EBGM05 = 5.22 vs. *n* = 103, EBGM05 = 2.38 vs. *n* = 268, EBGM05 = 3.88) and investigations (*n* = 1,895, EBGM05 = 1.17 vs. *n* = 166, EBGM05 = 1.42 vs. *n* = 770, EBGM05 = 4.15). Furthermore, disproportionate reporting was observed for surgical and medical procedures (*n* = 121, EBGM05 = 0.29) and pregnancy, puerperium and perinatal conditions (n = 79, EBGM05 = 0.6) at the SOC level. These represent potential signals that are not currently labeled in the prescribing information for spironolactone, although this analysis cannot determine causality. Additionally, surgical and medical procedures (*n* = 12, EBGM05 = 0.3) and pregnancy, puerperium, and perinatal conditions (*n* = 2, EBGM05 = 0.07) represent new AEs for eplerenone. The 95% CI for the ROR only shows the lower limit of the 95% two-sided CI of the ROR.

### 3.3 AE analysis at the PT level

This investigation incorporated multiple statistical measures, including ROR, PRR, EBGM, and BCPNN, to evaluate the strength of these signals. This comprehensive approach enabled a thorough assessment of the data, thereby strengthening the credibility of the findings. Tables 2, 3 show the top 10 signal strengths of AEs of spironolactone, eplerenone, and finerenone at the preferred term (PT) level, ranked by cases and EBGM. We compared these with the adverse reactions spelled out in the drug instructions.

**Table 2:** Top 10 signal strength on the PT level associated with spironolactone, eplerenone, and finerenone from the FAERS database ranked by number of cases (n) (2004 Q1 to 2025 Q3).

PT	SOC	Cases	EBGM (EBGM05)
Spironolactone (sorted by cases)			
Hyperkalaemia	Metabolism and nutrition disorders	1,204	85.8 (80.88)
Acute kidney injury	Renal and urinary disorders	1,018	12.83 (12.04)
Drug interaction	General disorders and administration site conditions	627	9.67 (8.93)
Hyponatraemia	Metabolism and nutrition disorders	438	19.17 (17.43)
Hypotension	Vascular disorders	389	4.78 (4.32)
Dehydration	Metabolism and nutrition disorders	353	6.44 (5.79)
Drug hypersensitivity	Immune system disorders	257	3.28 (2.9)
Gynaecomastia	Reproductive system and breast disorders	255	17.86 (15.78)
Bradycardia	Cardiac disorders	176	8.09 (6.97)
Confusional state	Psychiatric disorders	171	2.58 (2.22)
Eplerenone (sorted by cases)			
Acute kidney injury	Renal and urinary disorders	49	9.56 (7.19)
Hyperkalaemia	Metabolism and nutrition disorders	29	32 (22.16)
Drug interaction	General disorders and administration site conditions	20	4.78 (3.07)
Renal failure	Renal and urinary disorders	19	5.31 (3.38)
Blood creatinine increased	Investigations	15	8.61 (5.18)
Cardiac failure	Cardiac disorders	12	5.68 (3.22)
Hyponatraemia	Metabolism and nutrition disorders	11	7.45 (4.12)
Cardiac disorder	Cardiac disorders	11	4.34 (2.4)
Dysphagia	Gastrointestinal disorders	10	3.83 (2.06)
Arrhythmia	Cardiac disorders	9	7.06 (3.67)
Finerenone (sorted by cases)			
Glomerular filtration rate decreased	Investigations	201	375.77 (325.09)
Hyperkalaemia	Metabolism and nutrition disorders	164	105.39 (89.97)
Blood creatinine increased	Investigations	150	50.16 (42.55)
Blood potassium increased	Investigations	105	144.72 (119)
Renal impairment	Renal and urinary disorders	89	23.72 (19.2)
Dizziness	Nervous system disorders	64	2.86 (2.23)
Acute kidney injury	Renal and urinary disorders	53	6.02 (4.59)
Urine albumin/creatinine ratio increased	Investigations	43	2022.32 (1470.24)
Hypotension	Vascular disorders	40	4.43 (3.24)
Hyponatraemia	Metabolism and nutrition disorders	29	11.44 (7.94)

EBGM, Empirical Bayes Geometric Mean; PT, preferred terms; SOC, system organ classes.

**Table 3:** Top 10 signal strength on the PT level associated with Spironolactone, eplerenone and finerenone from the FAERS database ranked by EBGM (2004 Q1 to 2025 Q3).

PT	SOC	Cases	EBGM (EBGM05)
Spironolactone (sorted by EBGM)			
Endometriosis male	Reproductive system and breast disorders	7	1,802.53 (221.76)
Secondary sexual characteristics absence	Endocrine disorders	3	618.01 (159.8)
Blood aldosterone abnormal	Investigations	3	386.26 (110.06)
Double hit lymphoma	Neoplasms benign, malignant and unspecified (incl cysts and polyps)	5	257.5 (100.88)
Female sexual arousal disorder	Reproductive system and breast disorders	8	196.19 (94.68)
Gender dysphoria	Psychiatric disorders	8	156.96 (76.32)
Asthenospermia	Reproductive system and breast disorders	4	132.91 (48.25)
Mucous membrane pemphigoid	Skin and subcutaneous tissue disorders	4	117.72 (42.9)
Blood aldosterone increased	Investigations	8	111.35 (54.6)
Spur cell anemia	Blood and lymphatic system disorders	3	108.42 (33.9)
Eplerenone (sorted by EBGM)			
Peripheral artery thrombosis	Vascular disorders	3	58.57 (18.85)
Weight abnormal	Investigations	3	47.93 (15.43)
Hyperkalaemia	Metabolism and nutrition disorders	29	32 (22.16)
Cardiac failure chronic	Cardiac disorders	3	27.09 (8.72)
Cutaneous vasculitis	Skin and subcutaneous tissue disorders	3	23.33 (7.51)
Blood potassium increased	Investigations	8	18.94 (9.45)
Polyuria	Renal and urinary disorders	4	18.55 (6.95)
Blood pressure inadequately controlled	Vascular disorders	3	17.96 (5.78)
Purpura	Skin and subcutaneous tissue disorders	4	17.2 (6.44)
Pemphigoid	Skin and subcutaneous tissue disorders	3	16.29 (5.25)
Finerenone (sorted by EBGM)			
Urine albumin/creatinine ratio increased	Investigations	43	2,022.32 (1470.24)
Urine albumin/creatinine ratio abnormal	Investigations	4	940.61 (343.8)
Albuminuria	Renal and urinary disorders	12	505.5 (284.49)
Albumin urine present	Investigations	7	414.14 (195.6)
Glomerular filtration rate decreased	Investigations	201	375.77 (325.09)
Blood potassium increased	Investigations	105	144.72 (119)
Microalbuminuria	Renal and urinary disorders	4	111.74 (41.78)
Hyperkalaemia	Metabolism and nutrition disorders	164	105.39 (89.97)
Blood potassium abnormal	Investigations	6	50.92 (22.83)
Blood creatine increased	Investigations	10	50.37 (27.05)

EBGM, Empirical Bayes Geometric Mean; PT, preferred terms; SOC, system organ classes.

The top 3 frequent adverse safety signals associated with spironolactone were hyperkalemia (*n* = 1,204), acute kidney injury (*n* = 1,018), and drug interactions (*n* = 627). The three largest EBGM05 (the lower 5% credibility limit of the Empirical Bayes Geometric Mean) values were observed in cases of endometriosis in males (EBGM05 = 221.76, *n* = 7), absence of secondary sexual characteristics (EBGM05 = 159.8, *n* = 3) and abnormal blood aldosterone levels (EBGM05 = 110.06, *n* = 3). However, these extreme EBGM values are based on very small case numbers and should be interpreted with caution. Rare signals like these may be influenced by reporting bias, chance, or coding errors (e.g., incorrect gender or AE term selection). The same caution should be exercised when considering other rare, unlabeled signals, such as double hit lymphoma (*n* = 5, EBGM05 = 100.88) and female sexual arousal disorder (*n* = 8, EBGM05 = 94.68). These findings are hypothesis-generating only and do not confirm a causal association. The top 3 frequent adverse safety signals for eplerenone were acute kidney injury (*n* = 49), hyperkalaemia (*n* = 29), and drug interaction (*n* = 20); the top 3 largest EBGM05 values were hyperkalaemia (EBGM05 = 22.16), peripheral artery thrombosis (EBGM05 = 18.85), and weight abnormal (EBGM05 = 15.43). The instructions did not mention the following adverse signals: gynaecomastia (*n* = 8, EBGM05 = 4.33), acute hepatic failure (*n* = 3, EBGM05 = 2.7), pemphigoid (*n* = 3, EBGM05 = 5.25). The top 3 frequent adverse safety signals for finerenone were glomerular filtration rate decreased (*n* = 201), hyperkalaemia (*n* = 164), and blood creatinine increased (*n* = 150); the two largest EBGM05 values were an increased urine albumin/creatinine ratio (EBGM05 = 1,470.24, *n* = 43) and an abnormal urine albumin/creatinine ratio (EBGM05 = 343.8, *n* = 4). While these extreme values reflect the specific laboratory monitoring protocol for finerenone in diabetic nephropathy trials, the very small case numbers for some of these parameters (e.g., *n* = 4 for ‘abnormal’) warrant cautious interpretation. These signals should be considered exploratory rather than definitive safety findings. Our data mining revealed disproportionate reporting of several AEs not explicitly mentioned in the finerenone specifications, including acute kidney injury (*n* = 53, EBGM = 4.59), renal failure (*n* = 22, EBGM = 2.35), dizziness (*n* = 64, EBGM = 2.23), and hypokalemia (*n* = 9, EBGM = 3.42). The real-world study analysis based on the FAERS database also provides valuable reference information for revising the instructions for spironolactone, eplerenone, and finerenone.

### 3.4 Comparative analysis of clinical priorities

An analysis of the pharmacovigilance signals for the three MRA (spironolactone, eplerenone, and finerenone) reveals their distinct and shared safety profiles, which are based on clinical priority scoring derived from four signal recognition methods (Table 4).

**Table 4:** The clinical relevance of DME ranked as moderate clinical priorities associated with spironolactone, eplerenone and finerenone from the FAERS database (2004 Q1 to 2025 Q3).

PTs	Number of cases	Reporting rate (cases/non-cases)	Signal stability	Case fatality rate (%)	Clinical relevance	Clinical priorities (total score)
Spironolactone						
Acute kidney injury	1,018	4.20%	4 of 4	5.99%	DME	Moderate priority (5)
Toxic epidermal necrolysis	28	0.11%	4 of 4	50.00%	DME	Moderate priority (5)
Renal failure	161	0.64%	4 of 4	10.56%	DME	Moderate priority (4)
Agranulocytosis	38	0.15%	4 of 4	7.89%	DME	Moderate priority (4)
Erythema multiforme	14	0.06%	4 of 4	0.00%	DME	Moderate priority (4)
Dermatitis exfoliative generalized	7	0.03%	4 of 4	0.00%	DME	Moderate priority (4)
Eplerenone						
Renal failure	19	1.18%	4 of 4	5.26%	DME	Moderate priority (5)
Acute kidney injury	49	3.10%	4 of 4	2.04%	DME	Moderate priority (5)
Acute hepatic failure	3	0.18%	4 of 4	0.00%	DME	Moderate priority (4)
Finerenone						
Acute kidney injury	53	1.93%	4 of 4	1.89%	DME	Moderate priority (5)
Renal failure	22	0.79%	4 of 4	13.64%	DME	Moderate priority (4)

DME, designated medical event; PT, preferred terms.

The scope and diversity of AE signals vary significantly among MRA. Spironolactone was found to have the broadest profile in this dataset., with over 100 preferred terms spanning multiple SOC. These include not only anticipated renal, metabolic, and cardiovascular events but also serious cutaneous reactions such as toxic epidermal necrolysis, congenital malformations, and hormonal or sexual disorders. Eplerenone presents a more focused, though clinically severe, signal profile, predominantly featuring serious cardiac events like sudden death and cardiogenic shock, alongside renal and hepatic failure, with fewer distinct signals overall. In contrast, finerenone’s AEs are largely confined to the renal and metabolic domains, frequently including renal impairment, hyperkalemia, and related laboratory abnormalities such as decreased glomerular filtration rate and elevated creatinine. Among the three agents, finerenone exhibits the most limited range of system organ class involvement.

Spironolactone showed disproportionate reporting for several high-priority AEs, including congenital disorders such as gastrointestinal malformation and hypospadias, along with serious conditions like acute pulmonary edema. These events demonstrate alarmingly high case fatality rates, ranging from 55.56 to 100%, highlighting significant teratogenic and toxicity risks. In comparison, eplerenone is associated with high-priority events such as sudden death, reported as fatal in 66.67% of cases. Finerenone shows a comparatively milder profile, with acute kidney injury being its sole high-priority event and a notably lower reported fatality rate of 1.89%.

Hyperkalemia is a dominant and consistent safety signal across all three drugs, consistently classified as an Important Medical Event (IME) with moderate priority (score 4). It represents the most frequently reported event for both spironolactone (1,204 cases) and finerenone (164 cases). Acute kidney injury and renal failure are also significant adverse outcomes and are designated as Designated Medical Events (DMEs). Drug interaction risks are notable for spironolactone (627 cases) and eplerenone (20 cases), underscoring the importance of managing concomitant therapies. Additionally, gynecomastia emerges as a prominent and high-frequency signal for spironolactone (255 cases), aligning with its known antiandrogenic properties. This effect is absent from the safety profiles of the more selective agents, eplerenone, and finerenone.

All analyzed signals for the three drugs demonstrated perfect stability (3 of 3), reflecting consistent detection across methodologies. Spironolactone exhibits a mix of Designated Medical Events (DMEs) and Important Medical Events (IMEs) among its high- and moderate-priority events, indicating a broad spectrum of serious adverse reactions. In contrast, eplerenone and finerenone show a higher proportion of events including several serious ones, classified as “none” in terms of specific DME/IME relevance within their lower-priority categories, with the exception of their core renal and cardiac events.

### 3.5 Network pharmacology findings

#### 3.5.1 Target acquisition

Following summarization and deduplication, the following targets were obtained: 445 for spironolactone, 117 for eplerenone, 12 for finerenone, 12,495 for acute kidney injury and 12,856 for liver damage. Following integration, 360 targets for the combination of spironolactone and acute kidney injury, 111 targets for the combination of eplerenone and acute kidney injury, and 12 targets for the combination of finerenone and acute kidney injury were generated.

#### 3.5.2 PPI network and hub target analysis

Three hundred sixty spironolactone – acute kidney injury targets, 111 eplerenone – acute kidney injury targets, and 12 finerenone – acute kidney injury targets were imported into the String database separately, with the biological species set as “*Homo sapiens*,” to obtain protein interaction networks (Supplementary Figure 2). Through topological parameter analysis in Cytoscape (version 3.9.0), the target colors were sorted from dark to light based on degree values (Supplementary Figure 3), and the top 10 targets were selected according to the size of adjacent degree centrality (Supplementary Figure 4). The PPI regression curves of spironolactone – acute kidney injury R=0.6988 , eplerenone – acute kidney injury R=0.563 , and finerenone – acute kidney injury R=0.6677 indicate a positive correlation trend between protein degree and adjacent degree centrality, suggesting that these targets may play a major role in drug-induced disease responses (Supplementary Figure 5).

#### 3.5.3 GO and KEGG enrichment analysis

**Figure 3:**
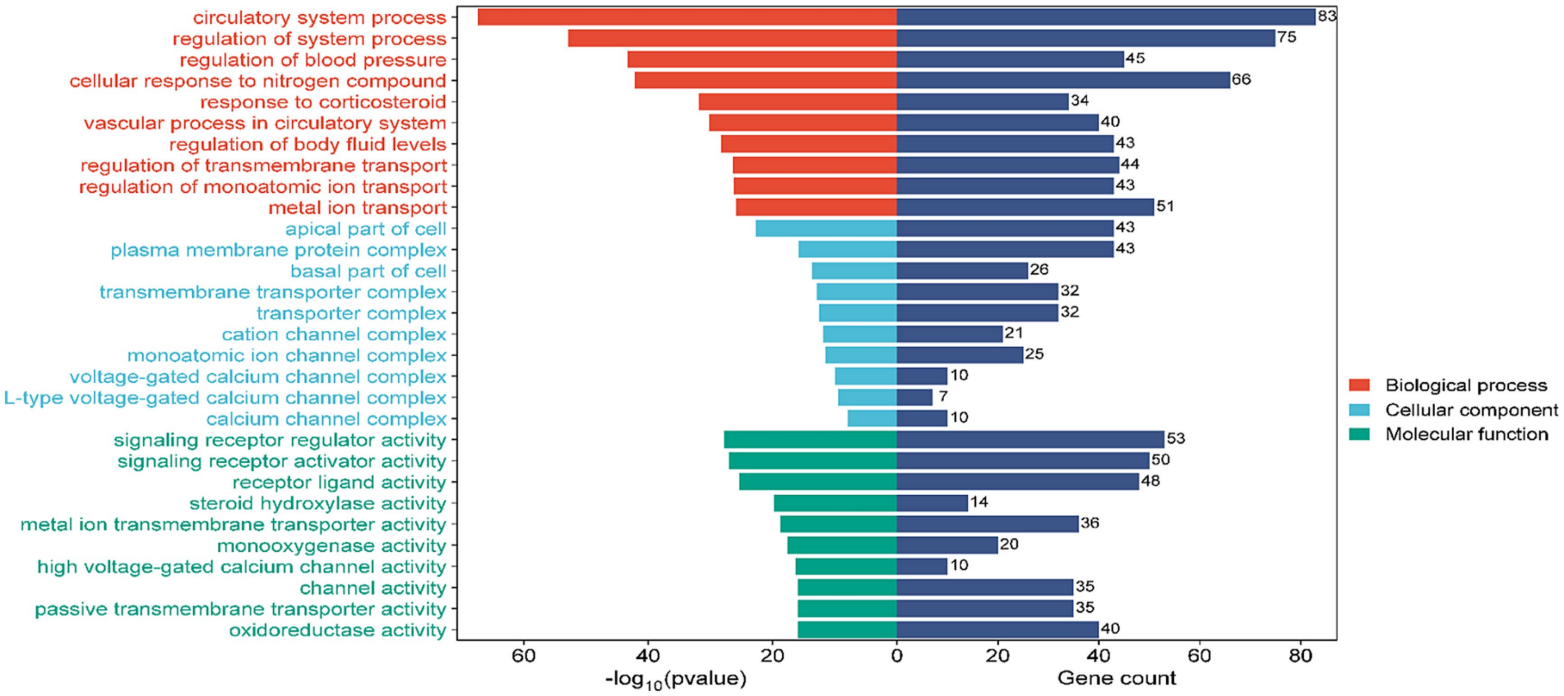
GO enrichment bar chart for spironolactone-acute kidney injury targets showing the top 10 biological processes, cellular components, and molecular functions based on *p*-value (DAVID database).

**Figure 4:**
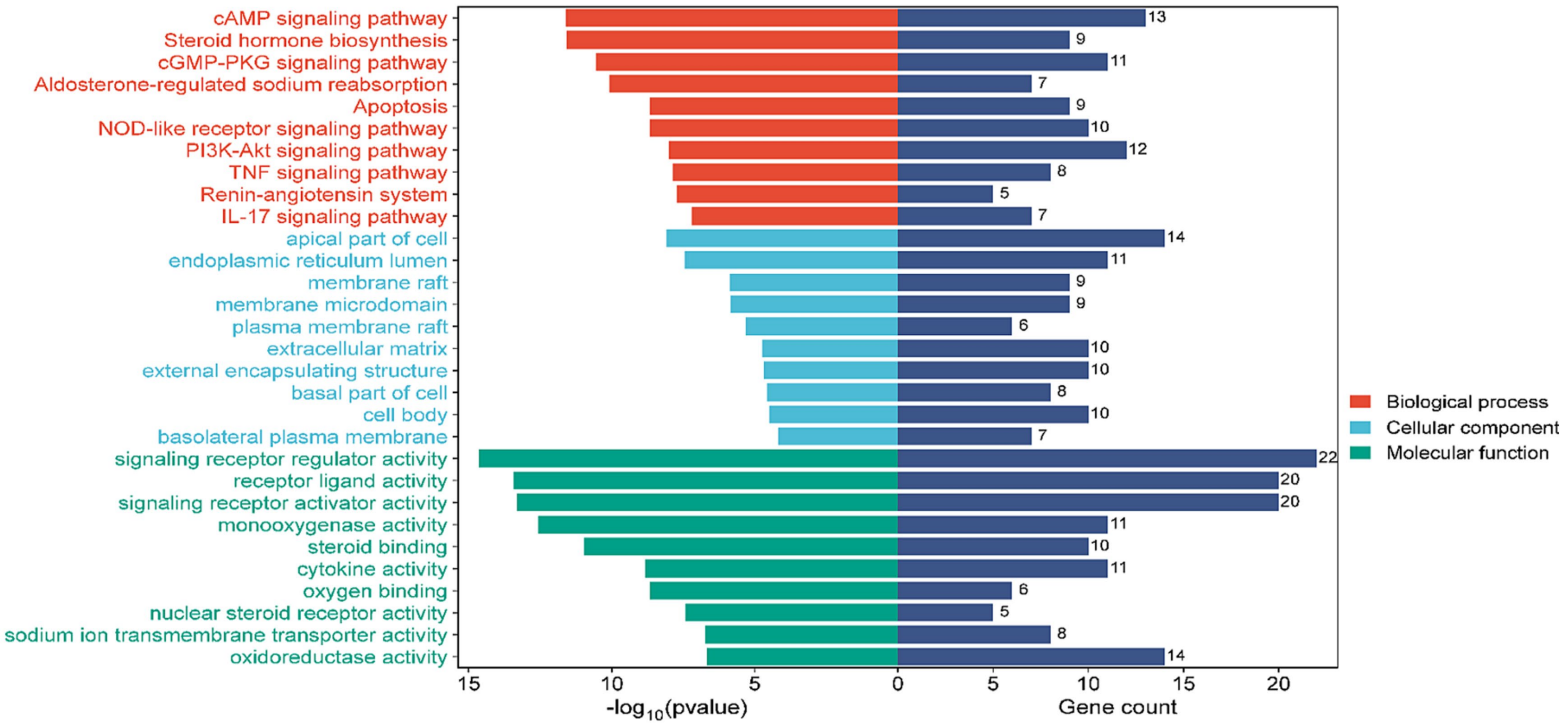
Enrichment analysis of eplerenone-acute kidney injury targets [top terms ranked by -log₁₀(*p*-value)]. The bar chart shows enriched terms across multiple categories, including KEGG pathways (e.g., cAMP signaling, cGMP-PKG signaling, steroid hormone biosynthesis), cellular components (e.g., apical part of cell, membrane raft), and molecular functions (e.g., receptor ligand activity, monooxygenase activity). Gene counts are indicated numerically. Analysis was performed using the DAVID database.

**Figure 5:**
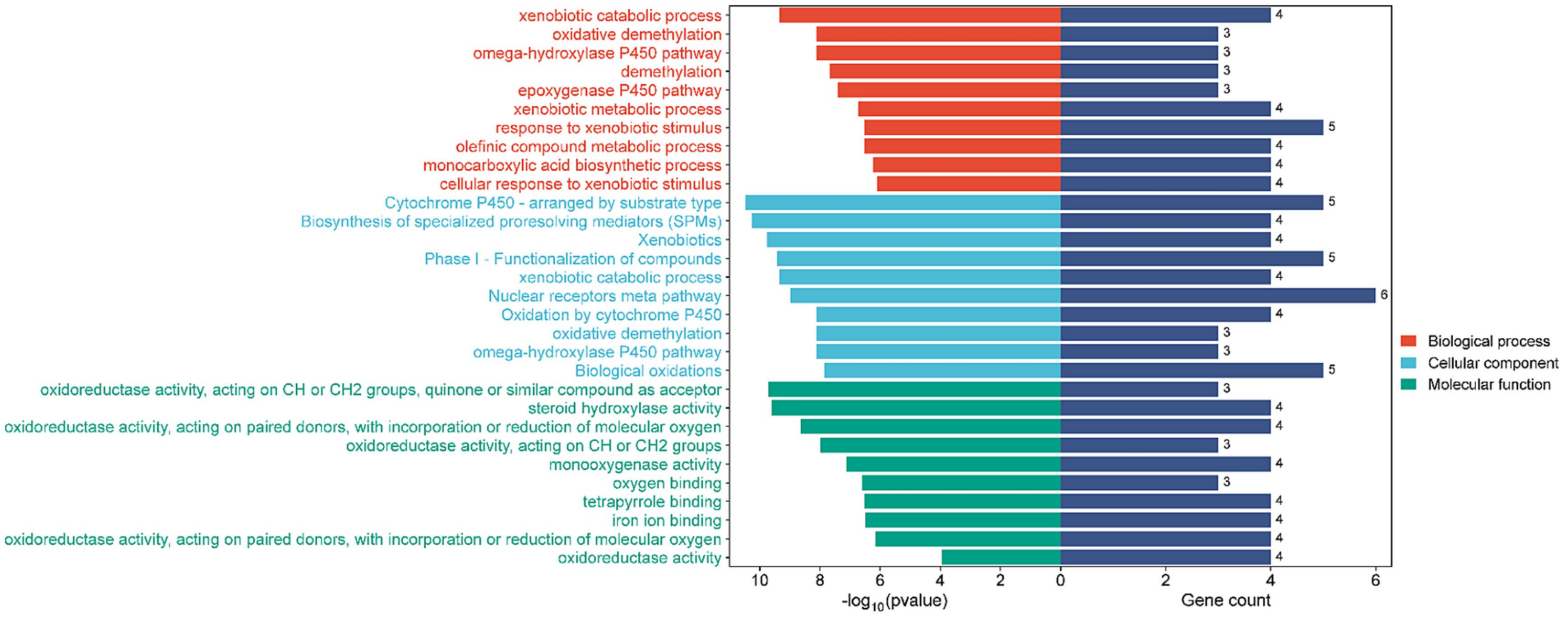
Functional enrichment analysis of finerenone-acute kidney injury targets. The bar chart displays the top enriched terms [ranked by -log_10_(*p*-value)] across biological processes (e.g., xenobiotic catabolic process, oxidative demethylation), molecular functions (e.g., steroid hydroxylase activity, monooxygenase activity, iron ion binding), and other relevant categories (e.g., cytochrome P450-related pathways). Analysis was performed using the DAVID database. Gene counts are shown numerically on the right. Due to the limited number of finerenone targets, the enrichment results likely reflect combined or expanded annotation sets.

Using the DAVID database, the targets of spironolactone, eplerenone, and finerenone – acute kidney injury were analyzed separately, and the top 10 most important terms were selected based on *p*-value size, generating GO enrichment analysis bar charts (Figures 3–5). The analysis indicated that these targets primarily participate in peptidase and peptidase receptor activity at the molecular level, with biological processes mainly manifesting as the regulation of protein catabolism. From a cellular composition perspective, these targets are mainly located within the cytoplasm and vesicular cavities. KEGG enrichment analysis identified the top 10 most important pathways for the reactions induced by the two aforementioned drugs, presented as Sankey-bubble diagrams (Supplementary Figures 6A–C). Spironolactone, eplerenone and finerenone induced acute kidney injury involve multiple targets and multiple signaling pathways.

## 4 Discussion

This study used the FAERS database to identify and compare adverse reaction signals for three MRA: spironolactone, eplerenone, and finerenone. Disproportionality analyses were performed using frequentist methods (ROR and PRR) and Bayesian methods (BCPNN and EBGM). Signals were defined as positive only if they were detected by all four algorithms. As the study design is retrospective and descriptive, all findings represent statistical associations rather than confirmed causal relationships. It is important to emphasize that disproportionality analysis in spontaneous reporting systems detects reporting imbalances, not causal effects. Therefore, all signals reported here should be interpreted as generating hypotheses, and any comparisons of signal strength between drugs should not be misconstrued as definitive rankings of comparative toxicity.

The study analyzed reports of AEs attributed to spironolactone, eplerenone, and finerenone use. The predominance of reports originating from the United States (35.1% versus 32.8% versus 68.4%) can be attributed to the country’s higher awareness of and participation in drug safety reporting among healthcare professionals and consumers. Additionally, the elderly population was the main reporting group, accounting for the majority of all reports (40.6, 39.0, 33.2% for patients aged 65–85 years), which is consistent with the epidemiological characteristics of patients with hypertension, HFrEF, CKD, and type 2 diabetes. As mineralocorticoids are increasingly used in clinical settings, clinicians must be aware of potential adverse reactions, especially in elderly patients. Since these reactions may be life-threatening or affect disease progression, it is crucial to recognize them early (19). The observed gender distribution—greater female reporting for spironolactone and male predominance for eplerenone and finerenone, partially reflects real-world prescribing patterns. Spironolactone is commonly prescribed to women for conditions such as polycystic ovary syndrome, acne, and hirsutism, in addition to its cardiovascular uses. This may explain why it is reported more frequently by women. In contrast, eplerenone, and finerenone are primarily prescribed for cardiorenal syndromes, which are more prevalent in men, especially older adults with diabetes or chronic kidney disease. This pattern aligns with epidemiological studies that describe sex differences in cardiorenal disease and drug utilization. Additionally, serious and important medical events were the most frequently reported outcomes associated with these drugs (58.0, 72.6, and 77.4%, respectively).

### 4.1 Insights from SOC level

Results indicate significant differences in the adverse reaction profiles of the three drugs: reports for spironolactone and eplerenone involved a broader range of SOCs, while reports for finerenone were primarily concentrated in a three number of SOCs.

Spironolactone has been on the market for over 60 years. Spironolactone is generally considered safe and well-tolerated, but an observational study of 788 hospitalized patients reported several adverse reactions. The most frequent adverse drug event (AE) was hyperkalemia, observed in 68 patients (8.6%). Other AEs included dehydration (3.4%), hyponatremia (2.4%), and gastrointestinal symptoms (2.3%), including loss of appetite, nausea, vomiting, and diarrhea. Neurological disorders (2.0%) were also reported, including headache, dizziness, tremors, and confusion. Skin rashes and male breast enlargement were observed in 10 patients (1.3%), while other AEs were reported less frequently (20). These AEs occurred more frequently in females, and the incidence rate was higher in the elderly population. This is largely due to the pronounced anti-androgenic effects of the drug when used to treat conditions such as acne, hirsutism, and androgenic alopecia in women (21, 22). Spironolactone is also commonly used in feminizing hormone therapy for individuals transitioning from male to female. Conversely, when used by men, spironolactone is more likely to induce feminizing side effects, such as gynecomastia. Its anti-androgenic activity is also less potent in men, which limits its use for male-related conditions (23). This study may have several AEs linked to spironolactone, including congenital, familial, and genetic disorders; ear and labyrinth disorders; and pregnancy, puerperium, and perinatal conditions. None of these are mentioned in the drug’s labeling. This underscores the need for more comprehensive drug labeling that better reflects potential AEs.

Eplerenone, an aldosterone antagonist, increases the survival rate of stable patients with symptomatic heart failure and a reduced ejection fraction (HFrEF) after an acute myocardial infarction. It was approved by the FDA in 2002. The four algorithms identified significant SOCs, including cardiac, renal, and urinary disorders, as well as metabolism and nutrition disorders. These disorders had been reported in clinical trials or shown in the drug label. AEs mentioned in the FDA’s drug label include hyperkalemia, increased creatinine, and hypertension. Our consistency analysis revealed that finerenone was associated with significant AEs in several SOCs, including investigations, metabolism and nutrition disorders, renal and urinary disorders, gastrointestinal disorders, and cardiac disorders. Within the investigations SOC, finerenone is generally associated with a decreased glomerular filtration rate, as well as increased creatinine or potassium in the blood. Common AEs related to metabolism and nutrition disorders included hyperkalemia, hyponatremia, and dehydration. This was also confirmed in our study, which also illustrated the reliability of our results.

### 4.2 Insights of safety risks

#### 4.2.1 Difference of core safety risks across drug comparisons

Based on the results derived from the FAERS database analysis, several notable similarities and distinctions emerge when compared with previous pharmacovigilance and clinical studies on MRAs.

Consistent with established literature, hyperkalemia and acute kidney injury remain predominant safety concerns across all three MRAs, particularly with spironolactone and eplerenone (24). Spironolactone is a potassium-sparing diuretic. Elevated potassium levels (hyperkalemia) are another possible side effect, especially in individuals with renal dysfunction or heart failure. This side effect is more common at higher dosages (25). According to the prescribing information, routine monitoring of potassium levels is recommended for patients taking spironolactone for Food and Drug Administration (FDA)-approved uses, such as treating hypertension and heart failure (26). According to the most recent prescribing information from 2018, potassium levels should be monitored within 1 week of initiating or titrating spironolactone. Then, monitoring should occur monthly for the first 3 months, quarterly for the following year, and subsequently every 6 months (27). In earlier clinical studies and a randomized controlled trial, the most frequently reported AEs of eplerenone for HFrEF were hyperkalemia and increased blood creatinine, which are also listed in the prescribing information for eplerenone (28). This aligns with their known pharmacologic action of inhibiting aldosterone, which can impair renal potassium excretion and, in susceptible patients, and associated with renal hypoperfusion or injury. The higher frequency of hyperkalemia with spironolactone (*n* = 1,204) corroborates findings from large – scale observational studies and clinical trials, such as those in heart failure and resistant hypertension populations, where spironolactone use is frequently associated with electrolyte disturbances (29). Notably, although the absolute number of finerenone reports exceeded that of eplerenone, its signal strength was relatively low. Patients with CKD and type 2 diabetes are at an increased risk for hyperkalemia due to decreased renal function, an expected side effect of MR antagonism. Therefore, potassium levels in the blood should be closely monitored during finerenone treatment. Similarly, the strong hyperkalemia signal for finerenone is consistent with its mechanism and has been highlighted in recent phase III trials (e.g., FIDELIO-DKD and FIGARO-DKD), albeit with a reportedly lower risk compared to older MRAs.

#### 4.2.2 Sex hormone-related AEs

This study provides real-world evidence that supports the well-known link between spironolactone and sex hormone-related AEs: both the number of gynaecomastia reports (*n* = 255) and signal strength (EBGM05 = 15.78) were significantly higher than those for eplerenone (*n* = 8, EBGM05 = 4.33). Additionally, spironolactone exhibited rare yet highly specific signals including “male endometriosis” (EBGM05 = 221.76) and “female sexual arousal disorder” (EBGM05 = 94.68).

Notably, the signal for gynecomastia with spironolactone (*n* = 255) is well-documented in prior literature due to its antiandrogenic activity, and its absence in eplerenone and finerenone profiles supports their higher receptor selectivity, a pharmacological advantage previously reported in preclinical and clinical comparisons (30). Similarly, the broader range of systemic AEs associated with spironolactone, including endocrine, cutaneous, and congenital disorders, has been described in earlier safety reviews and label updates, highlighting its pleiotropic off-target effects.

In addition to gynecomastia, we found that erectile dysfunction may develop in the reproductive system and associated with breast diseases. A 2003 study by William F. Young, Jr., reported that eplerenone is a selective aldosterone receptor blocker that does not have the side effects associated with spironolactone, such as gynecomastia, erectile dysfunction, and menstrual irregularities. However, our research showed that aldosterone receptor blockade may only partially reverse these effects. This suggests that eplerenone may still be associated with side effects (31). Therefore, these side effects should be closely monitored during eplerenone use, as they may affect patient compliance.

The above data perfectly corroborates the pharmacological distinctions: Spironolactone, as a non-selective steroid MRA, exhibits antiandrogenic and progestogenic activity that leads to widespread endocrine side effects. Eplerenone, with its improved selectivity, significantly reduces these risks, although a small number of reports were still observed in our analysis. As a non-steroidal agent, finerenone mechanistically avoids such interference. This provides critical guidance for clinical selection: For patients requiring long-term therapy or those sensitive to endocrine side effects, finerenone, or eplerenone should be prioritized.

#### 4.2.3 Renal safety

Acute kidney injury and renal failure are nephrotoxicity events commonly reported across all three drug classes. The high frequency of reports for “decreased glomerular filtration rate” (*n* = 201) and “elevated serum creatinine” (*n* = 150) associated with finerenone differs from the patterns observed with spironolactone and eplerenone.

This does not imply that finerenone exhibits greater nephrotoxicity, but rather reflects its unique clinical application and monitoring paradigm: as a renal protective agent for diabetic nephropathy, its target patient population already presents with impaired renal function. Treatment necessitates rigorous monitoring of eGFR and serum creatinine as efficacy and safety indicators, consequently elevating the reporting rate of related laboratory abnormalities. This suggests that clinicians should accurately distinguish between drug-induced transient renal function changes and disease progression when managing finerenone.

#### 4.2.4 Caveats on the interpretation of rare signals

Some of the signals identified in this study, such as ‘endometriosis in males’ (*n* = 7) and ‘double hit lymphoma’ (*n* = 5), exhibited extreme EBGM values, but these were based on very small case numbers. These signals should not be interpreted as established safety findings. In the case of ‘endometriosis in males,’ the most plausible explanation is a reporting error, such as incorrect selection of the gender field (male) or the AE term (endometriosis), rather than a genuine drug–event association. While a single case report has described endometriosis in a man receiving hormonal therapy for prostate cancer (32). Two cases were also reported in the WHO VigiAccess, though there was no strong EBGM signal. It was reported that the use of spironolactone was associated with a hormonal state that could be linked to endometriosis in this 52-year-old man (33). Lin et al. (34) also referenced these AEs in FDA pharmacovigilance studies such occurrences are exceedingly rare and not generalizable. Therefore, we emphasize that rare and biologically questionable signals derived from spontaneous reporting systems must be interpreted with extreme caution and should not be given the same weight as robust and clinically plausible signals (e.g., hyperkalemia, acute kidney injury and gynaecomastia). These findings are hypothesis-generating only.

### 4.3 Placing research findings within a broader clinical context

Compared to earlier pharmacovigilance studies, the present analysis employs a more granular, multi-method signal detection approach (ROR, PRR, EBGM, and BCPNN), thereby enhancing the robustness of the findings. Elevated EBGM signals for rare events such as “endometriosis male” associated with spironolactone or “urine albumin/creatinine ratio abnormal” with finerenone though derived from limited cases (Table 3), highlight potential areas warranting further investigation, some of which have not been sufficiently emphasized in prior literature.

This study provides real-world evidence to inform clinical decision-making. For example, eplerenone may serve as a lower-risk alternative for male patients with heart failure who experience adverse endocrine effects from spironolactone. For patients with diabetic nephropathy, finerenone offers considerable advantages by providing cardiorenal protection without sex hormone–related side effects. However, enhanced monitoring of serum potassium and renal function is necessary.

Potential risk signals that are not currently reflected in drug labels were also identified. These include “double-hit lymphoma” and “female sexual arousal disorder” associated with spironolactone; “acute liver failure” and “bullous pemphigoid” related to eplerenone; and “hypokalemia” and “dizziness” linked to finerenone. These findings provide data that could inform updates to prescribing information and the issuance of safety alerts. They also alert clinicians to maintain vigilance.

Drug interactions were among the top three AE signals for both spironolactone and eplerenone. This suggests that using these drugs with renin-angiotensin system inhibitors, potassium-sparing diuretics, and other relevant agents may increase the risk of hyperkalemia and renal impairment. This underscores the need for more intense monitoring.

In summary, these findings reinforce the established safety profiles of MRAs and reveal less-documented associations in real-world settings. The findings confirm the broader AE spectrum of spironolactone compared to its newer counterparts and support the differentiated safety profiles of eplerenone and finerenone. Incorporating these real-world signals into future label updates and clinical monitoring protocols could be beneficial, especially regarding underreported organ systems and unlisted AEs.

### 4.4 Integrating network pharmacology with pharmacovigilance signals

The network pharmacology analysis provided molecular-level insights that complemented our findings based on the FAERS database. For example, hyperkalemia and acute kidney injury, which were dominant signals for all three MRAs in the real-world data, were found to be linked to shared targets, such as the mineralocorticoid receptor (NR3C2) and renal ion transporters. This was revealed by KEGG pathways, including ‘aldosterone-regulated sodium reabsorption’ and ‘collecting duct acid secretion’. The high-degree hub targets identified (e.g., EGFR, MAPK1 and SRC) are involved in inflammatory and fibrotic pathways, which is consistent with the known renal effects of MRAs. Interestingly, finerenone exhibited the fewest AKI-related targets n=12 , yet still displayed a robust clinical signal for acute kidney injury (EBGM05 = 4.59, indicating moderate priority), suggesting that its more limited target profile does not necessarily equate to reduced renal risk in real-world practice. Conversely, spironolactone’s extensive target network (360 targets) aligns with its broader range of renal and electrolyte AEs. Converging network pharmacology and pharmacovigilance strengthens the evidence base for the distinct safety profiles of the three MRAs, underscoring the value of integrating computational target prediction with real-world AE monitoring. These *in silico* findings are exploratory and do not confirm causality. These *in silico* findings are exploratory and do not confirm causality. They are intended to generate hypotheses that can be tested in future experimental studies.

### 4.5 Research limitations

Although this study identified many AEs that are not currently of concern, there are inevitably some limitations to the study. First, the FAERS database is a spontaneous reporting database, such as underreporting and selective reporting, which means the database may not be complete or fully representative of real-world adverse reaction rates. Second, the drug-related AEs obtained using these four algorithms only demonstrate correlation; a direct causal relationship between the drug and the adverse reaction requires further research. Although multiple signal detection algorithms were employed, signals do not equate to causality but merely suggest potential associations. The differences in reported case numbers observed in this study (e.g., spironolactone significantly more than the other two drugs) are primarily influenced by factors such as drug launch timing, patient usage volume, and clinical attention levels. These differences cannot be directly equated to variations in actual incidence rates. The findings still require validation through prospective studies. Third, while the FDA analyzes all collected adverse drug events, it is difficult to determine a direct causal relationship with a specific drug, as this may be confounded by other interacting drugs. Fourth, while our study identified several novel and high-priority AE signals (e.g., ‘endometriosis in males’ for spironolactone and ‘acute kidney injury’ for finerenone), these findings are derived from a spontaneous reporting system and represent statistical associations, not confirmed causality. The primary value of pharmacovigilance signal detection studies lies in identifying potential safety concerns that warrant further investigation, rather than providing definitive mechanistic proof; these studies are inherently hypothesis-generating. It is important to reiterate that disproportionality analysis identifies statistical associations, rather than causal relationships. Therefore, any suggestions for label revisions or clinical prioritization based solely on these findings are preliminary and require confirmation through well-designed observational or experimental studies. *In vitro* cytotoxicity or pathway assays would indeed be the ideal next step to test the biological plausibility of these signals. For instance, one could evaluate the impact of spironolactone on endometrial cell proliferation within a male hormonal environment or examine the effect of finerenone on renal tubular epithelial cell viability in a diabetic setting. We have provided network pharmacology data, which links the drugs to key AEs via known molecular pathways. Fifth, the FAERS database lacks standardized dosage information, which precludes formal dose- or exposure-response analysis. As a surrogate measure of exposure duration, we categorized high-priority AEs according to time-to-onset intervals. However, this approach cannot replace actual dose–response modeling or the determination of toxicological thresholds, such as IC₅₀ values. Consequently, the signals reported herein should be interpreted as hypothesis-generating, and future confirmatory studies, particularly *in vitro* dose–response experiments using relevant cell lines, are required to establish causal concentration-effect relationships. Sixth, particular caution should be exercised when interpreting rare signals with extreme disproportionality scores but very small case counts. Such signals are inherently unstable in spontaneous reporting systems and may arise from reporting bias, chance, or coding errors. They should not be overinterpreted or presented as being equivalent to well-established, clinically plausible signals. We have therefore reframed these findings as hypothesis-generating observations that require independent validation before any clinical or regulatory conclusions can be drawn. Finally, although FAERS is a widely used and accepted source for signal detection, it is subject to inherent biases, including under- and selective reporting, as well as a lack of denominator data. Therefore, all findings are hypothesis-generating. To assess the reproducibility and generalisability of the signals identified, independent validation in other spontaneous reporting systems is essential. For example, reports on finerenone are highly concentrated in the United States (68.4%), potentially influenced by its time to market and promotional activities. Several other limitations inherent should be also acknowledged, including indication bias arising from the confounding effect of underlying diseases, as well as the inability to adjust for comorbidities and concomitant medications due to the lack of detailed clinical data. Furthermore, inherent biases of spontaneous reporting systems may affect the reliability of the findings.

### 4.6 Future investigations

While this study provides a comprehensive signal landscape, it primarily identifies statistical associations within a spontaneous reporting system. Future research should pivot toward confirmatory and intervention studies that translate these signals into actionable clinical strategies. Pharmacists play a crucial role in proactively monitoring for drug-related adverse effects and providing comprehensive medication education to patients, thereby enhancing therapeutic safety and adherence (35–37). A critical and promising research direction lies in investigating the role of pharmacists in optimizing the safety of MRA therapy.

First, prospective, controlled studies are needed to validate the most significant unlabeled signals identified here, such as finerenone-associated acute kidney injury and spironolactone-related congenital disorders. Furthermore, research should focus on developing and testing pharmacist-led monitoring protocols tailored to the distinct risk profile of each MRA. For example, pharmacists could implement enhanced screening for hormonal effects and provide patient counseling on teratogenic risks for women of childbearing age taking spironolactone. For eplerenone and finerenone, they could emphasize closer monitoring of renal function and potassium levels, especially when initiating therapy and after dose adjustments. Second, the significant signal for drug interactions with spironolactone and eplerenone highlights an important area for pharmacist intervention. Future studies should design and evaluate the impact of pharmacist-driven medication review and reconciliation services for patients specifically prescribed MRAs. These services would aim to identify and mitigate the concurrent use of nephrotoxic agents, potassium-sparing drugs, and medications that inhibit MRA metabolism. This would help prevent adverse outcomes.

Finally, the underreporting inherent to FAERS underscores the necessity of structured surveillance. Research should explore integrating these pharmacovigilance signals into clinical decision support systems within pharmacy workflows. Evaluating the impact of real-time alerts for high-risk MRA profiles on pharmacist interventions, patient outcomes, and AE reporting rates would demonstrate the value of incorporating pharmacovigilance into routine pharmacy practice. Ultimately, empowering pharmacists as key agents in proactive MRA safety management could bridge the gap between signal detection and improved patient care.

## 5 Conclusion

This study analyzed real-world data to identify distinct safety signals for spironolactone, eplerenone, and finerenone. These signals should be treated as hypothesis-generating, they should not be interpreted as confirmed causal effects without further validation. Clinical selection should be based on individualized decision-making, taking into account the target disease, patient gender, baseline renal function, and serum potassium levels. Future mechanistic studies are urgently needed to validate the unlabeled signals, particularly: (1) *in vitro* renal tubular cell models to investigate finerenone-associated acute kidney injury via MAPK/PI3K-Akt pathways; (2) hormonal and endometrial cell proliferation assays to explore the association between spironolactone and “endometriosis in males”; and (3) comparative electrophysiological studies to elucidate the mechanisms underlying differential hyperkalemia across the three MRAs.

## Data Availability

The original contributions presented in the study are included in the article/Supplementary material, further inquiries can be directed to the corresponding authors.

## Footnotes

1
https://ctdbase.org/

2
http://stitch.embl.de/

3
https://prediction.charite.de/

4
http://www.swisstargetprediction.ch

5
https://lilab-ecust.cn/pharmmapper/index.html

6
https://www.omim.org/

7
https://www.genecards.org/

8
https://db.idrblab.net/ttd/

9
https://cn.string-db.org/

10
https://davidbioinformatics.nih.gov/

## Supplementary material

SUPPLEMENTARY FIGURE 1 Data filtering flowchart for adverse event reports of mineralocorticoid receptor antagonists (spironolactone, eplerenone, and finerenone) from the FAERS database (2004 Q1 to 2025 Q3). Stepwise filtering: (1) initial extraction using drug names as keywords; (2) deduplication based on CASEID and FDA_DT (keeping the latest ISR version); (3) retention of reports where the MRA was designated as the primary suspect (PS) drug; (4) exclusion of reports with missing critical data (e.g., no event PT). Final counts: spironolactone (*n* = 9,031), eplerenone (*n* = 646), finerenone (*n* = 1,879). FAERS, FDA Adverse Event Reporting System; ISR, Individual Safety Report; MRA, mineralocorticoid receptor antagonist; PS, primary suspect; PT, preferred term; SOC, System Organ Class.

SUPPLEMENTARY FIGURE 2 Protein-protein interaction networks of drug-AKI shared targets: **(A)** spironolactone-AKI (360 targets); **(B)** eplerenone-AKI (111 targets); and **(C)** finerenone-AKI (12 targets).

SUPPLEMENTARY FIGURE 3 PPI networks of drug-AKI shared targets with nodes colored by degree centrality. **(A)** Spironolactone-AKI, **(B)** eplerenone-AKI, and **(C)** finerenone-AKI.

SUPPLEMENTARY FIGURE 4 The top ten targets were ranked by degree centrality in the PPI networks: **(A)** spironolactone-AKI, **(B)** eplerenone-AKI and **(C)** finerenone-AKI.

SUPPLEMENTARY FIGURE 5 Regression curves of degree versus betweenness centrality in the PPI networks: **(A)** Spironolactone-AKI (*R* = 0.6988); **(B)** Eplerenone-AKI (*R* = 0.5631); and **(C)** Finerenone-AKI (*R* = 0.6677).

SUPPLEMENTARY FIGURE 6 KEGG enrichment Sankey‑bubble diagram for spironolactone, eplerenone, and finerenone‑AKI targets. Left: target‑pathway associations (bar width reflects number of pathways per target). Right: bubble plot with color indicating *P*‑value (red = more significant) and size indicating gene count. Top 10 pathways are presented.
