# Supplementary figures and images for: Pharmacovigilance profiles of three generations of mineralocorticoid receptor antagonists and network toxicology analysis
Source: Front Med (Lausanne). 2026 Jun 23;13:1797331. doi: 10.3389/fmed.2026.1797331 (PMC13337816; doi:10.3389/fmed.2026.1797331)

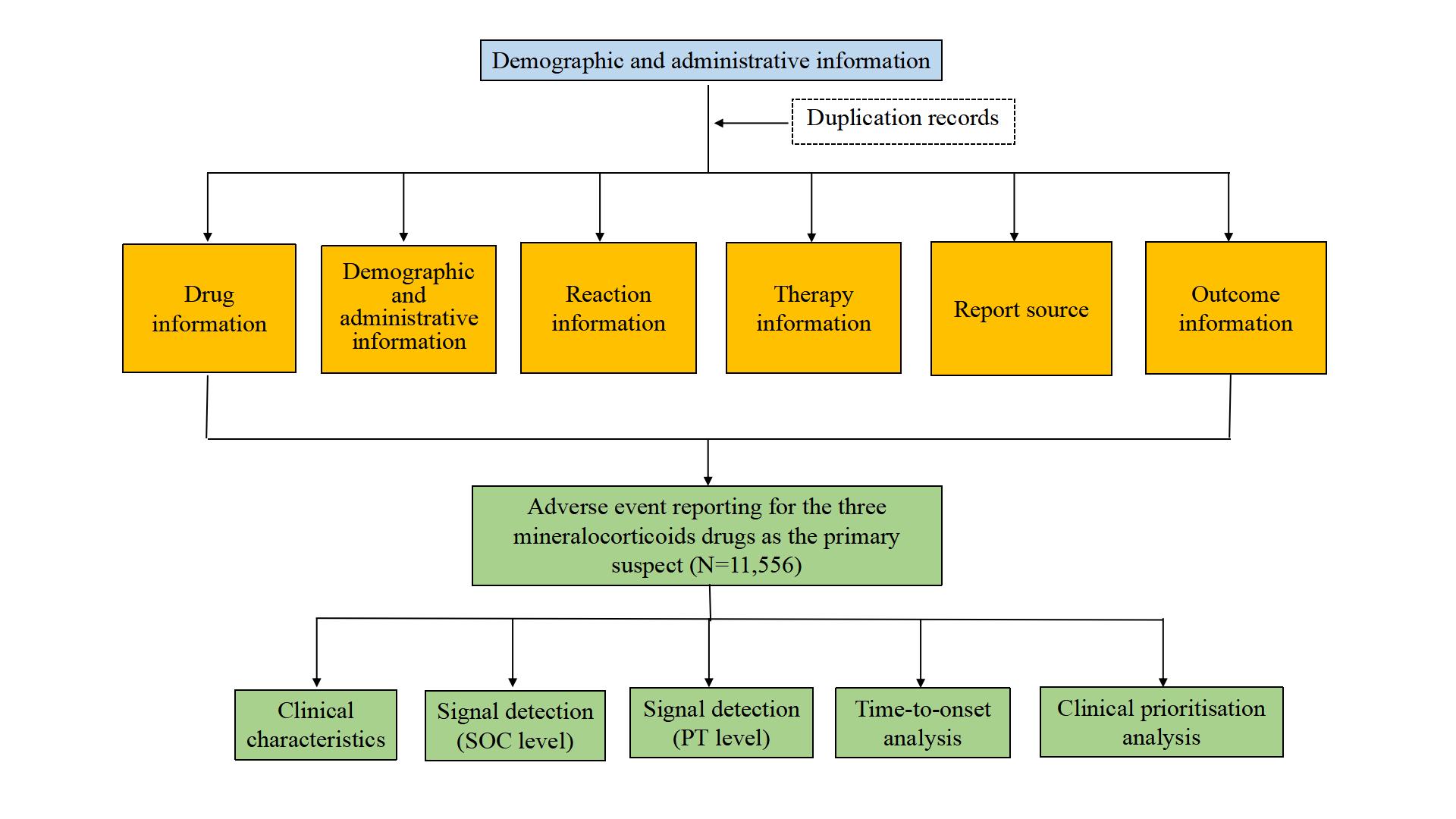

Supplement: SUPPLEMENTARY FIGURE 1 — Data filtering flowchart for adverse event reports of mineralocorticoid receptor antagonists (spironolactone, eplerenone, and finerenone) from the FAERS database (2004 Q1 to 2025 Q3). Stepwise filtering: (1) initial extraction using drug names as keywords; (2) deduplication based on CASEID and FDA_DT (keeping the latest ISR version); (3) retention of reports where the MRA was designated as the primary suspect (PS) drug; (4) exclusion of reports with missing critical data (e.g., no event PT). Final counts: spironolactone (n = 9,031), eplerenone (n = 646), finerenone (n = 1,879). FAERS, FDA Adverse Event Reporting System; ISR, Individual Safety Report; MRA, mineralocorticoid receptor antagonist; PS, primary suspect; PT, preferred term; SOC, System Organ Class. [file Image_1.jpeg]

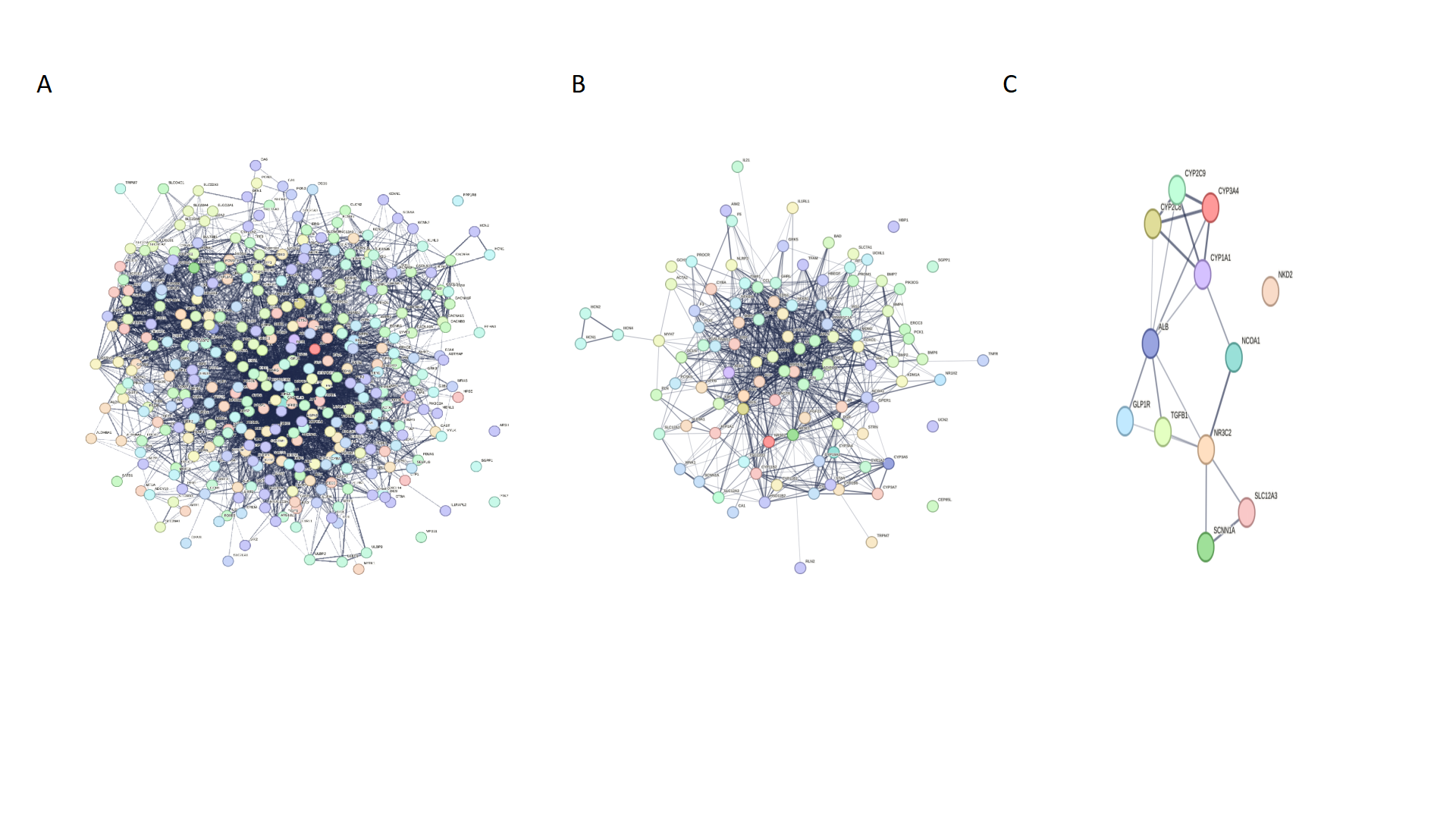

Supplement: SUPPLEMENTARY FIGURE 2 — Protein-protein interaction networks of drug-AKI shared targets: (A) spironolactone-AKI (360 targets); (B) eplerenone-AKI (111 targets); and (C) finerenone-AKI (12 targets). [file Image_2.tif]

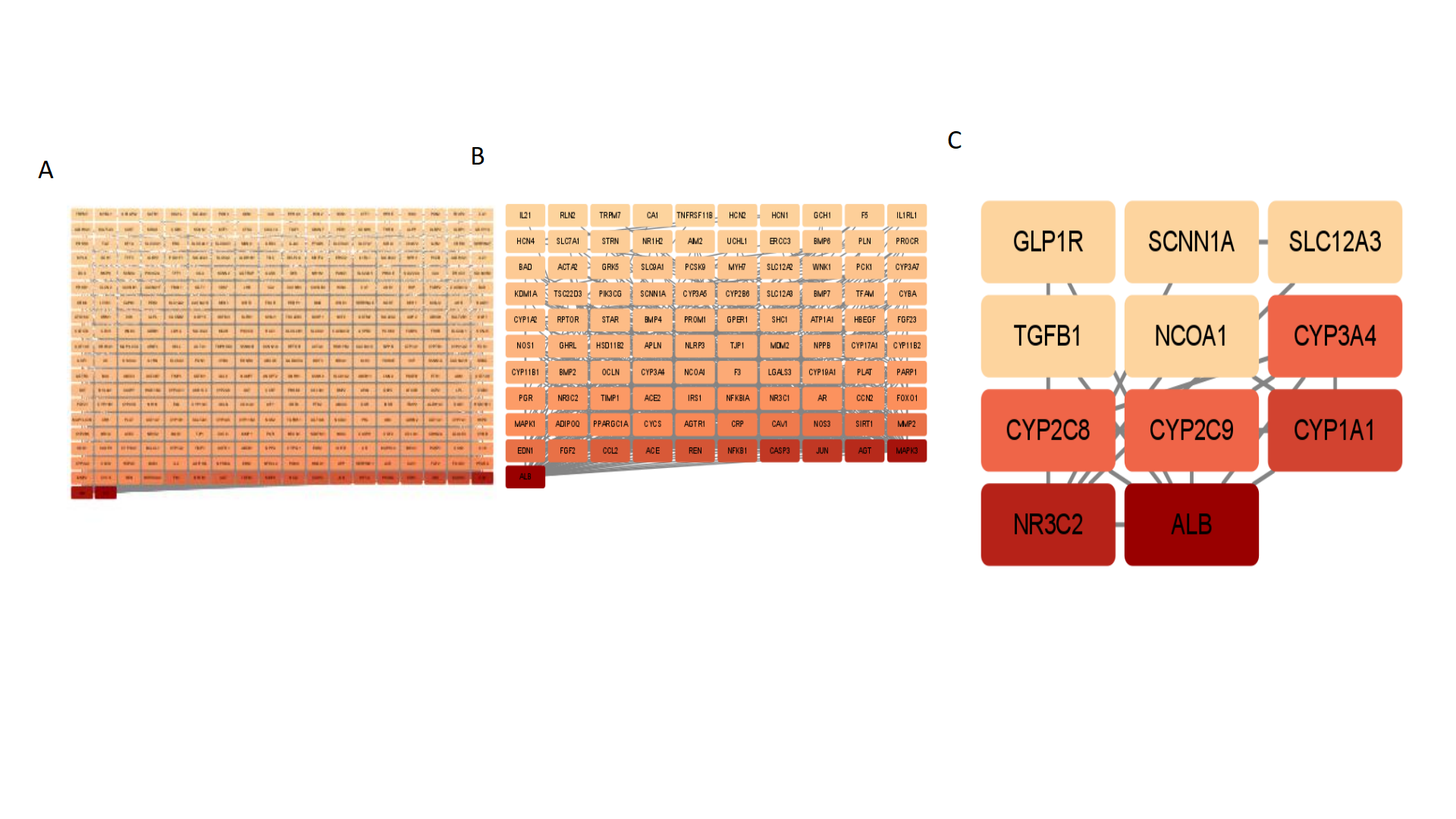

Supplement: SUPPLEMENTARY FIGURE 3 — PPI networks of drug-AKI shared targets with nodes colored by degree centrality. (A) Spironolactone-AKI, (B) eplerenone-AKI, and (C) finerenone-AKI. [file Image_3.tif]

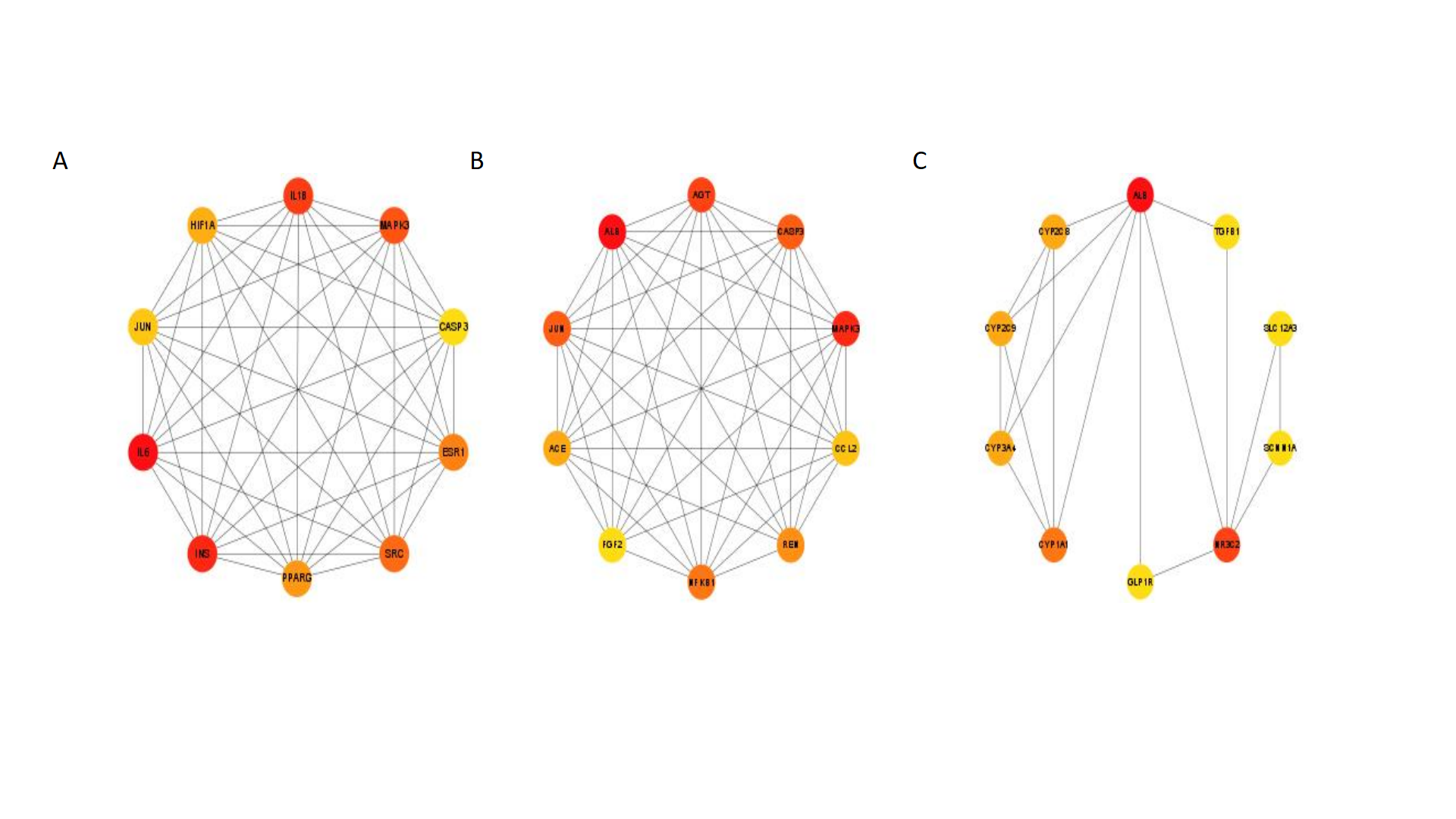

Supplement: SUPPLEMENTARY FIGURE 4 — The top ten targets were ranked by degree centrality in the PPI networks: (A) spironolactone-AKI, (B) eplerenone-AKI and (C) finerenone-AKI. [file Image_4.tif]

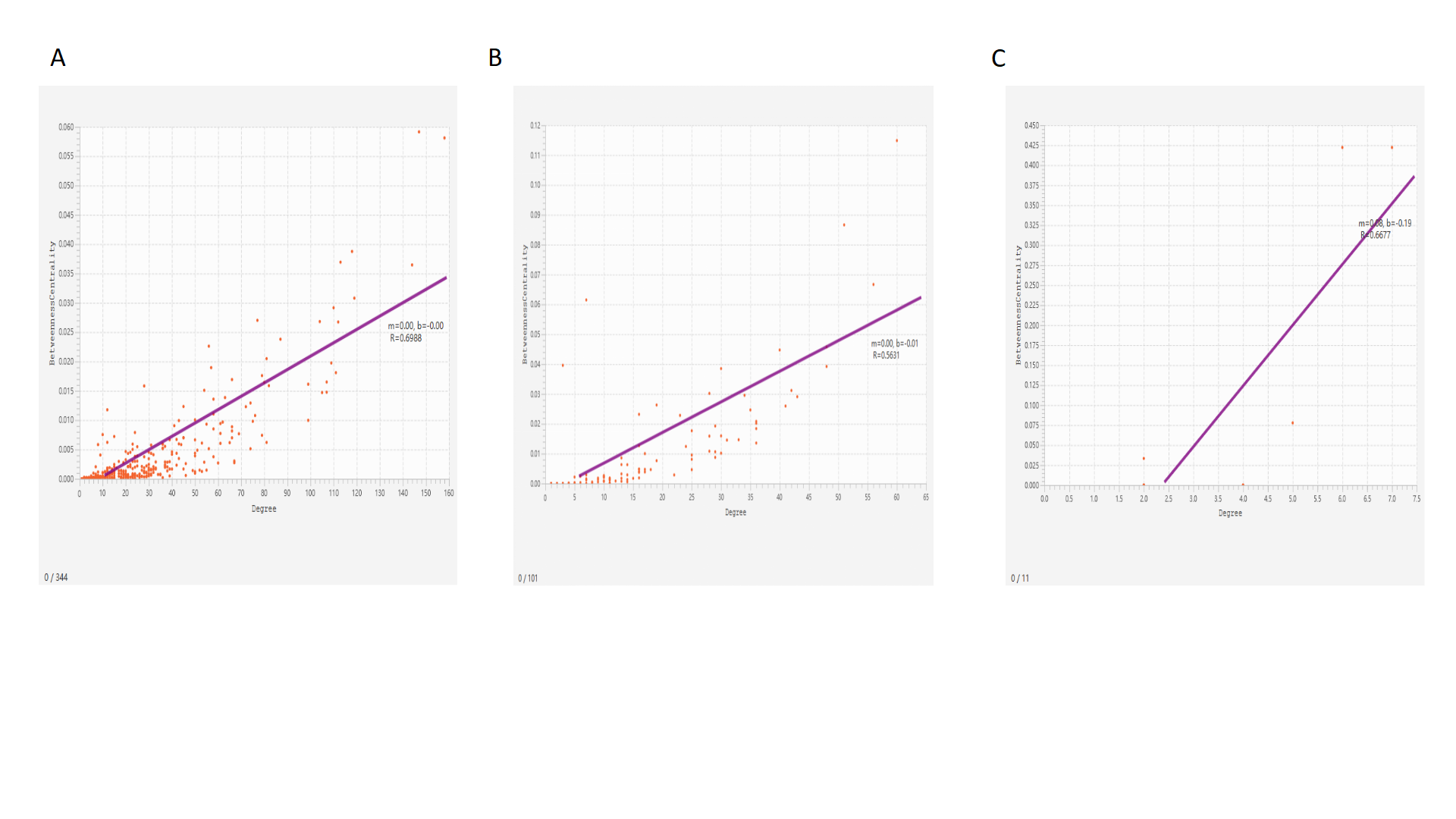

Supplement: SUPPLEMENTARY FIGURE 5 — Regression curves of degree versus betweenness centrality in the PPI networks: (A) Spironolactone-AKI (R = 0.6988); (B) Eplerenone-AKI (R = 0.5631); and (C) Finerenone-AKI (R = 0.6677). [file Image_5.tif]

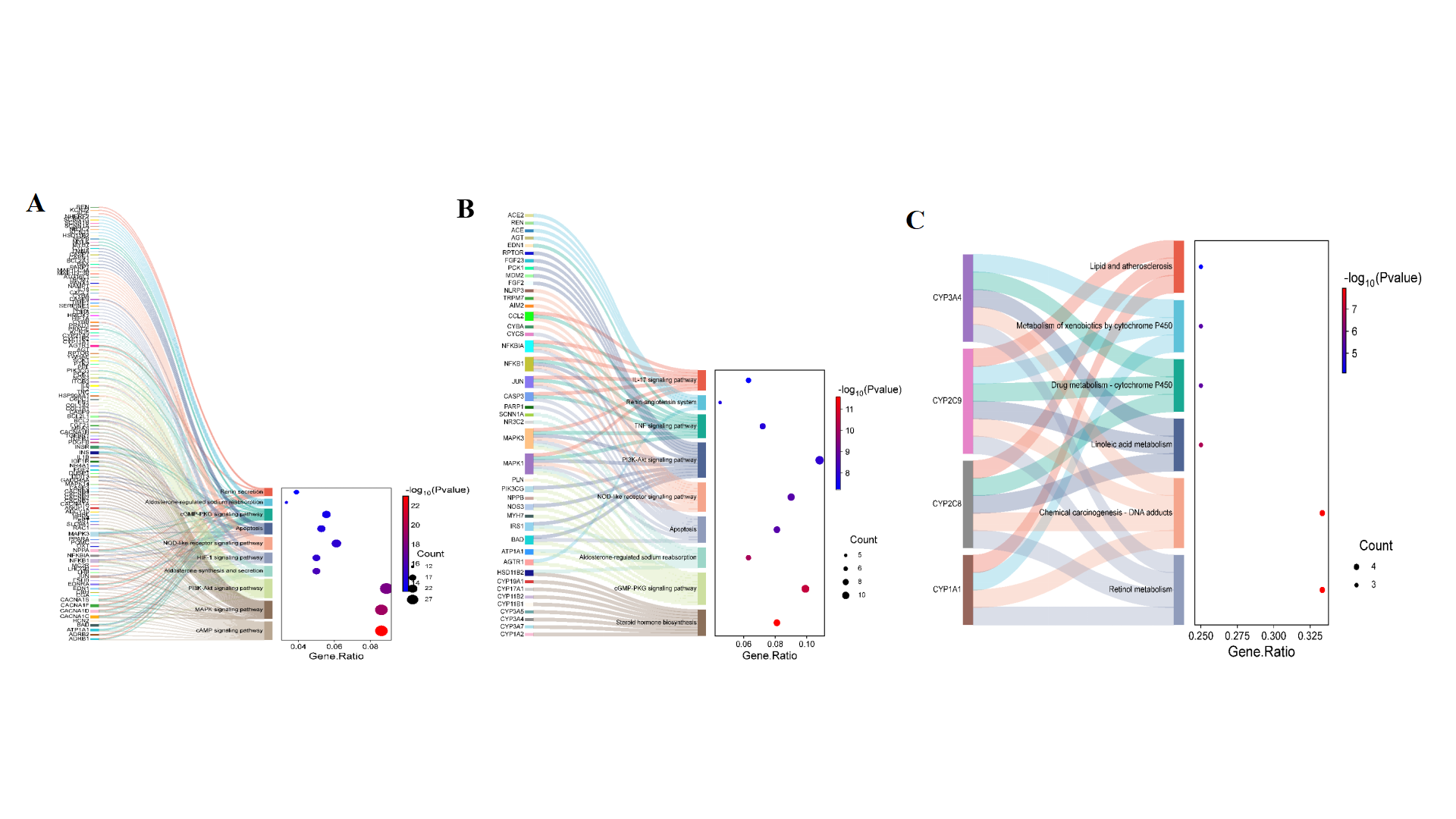

Supplement: SUPPLEMENTARY FIGURE 6 — KEGG enrichment Sankey‑bubble diagram for spironolactone, eplerenone, and finerenone‑AKI targets. Left: target‑pathway associations (bar width reflects number of pathways per target). Right: bubble plot with color indicating P‑value (red = more significant) and size indicating gene count. Top 10 pathways are presented. [file Image_6.tif]
