# Supplementary material for: Pharmacovigilance profiles of three generations of mineralocorticoid receptor antagonists and network toxicology analysis
Source: Front Med (Lausanne). 2026 Jun 23;13:1797331. doi: 10.3389/fmed.2026.1797331 (PMC13337816; doi:10.3389/fmed.2026.1797331)
Supplement: Supplementary file 7 [file Data_Sheet_1.docx]

**Supplementary Table 1.** Four grid table of the proportional imbalance methods.

| **Drug class** | **Number of reports of the target adverse events** | **Number of reports of the other adverse events** | **Total** |
| --- | --- | --- | --- |
| Ssuspect drug | a | b | a+b |
| Other drugs | c | d | c+d |
| Total | a+c | b+d | N=a+b+c+d |

Note: a: the number of reports containing both the suspect drug and the suspect adverse events; b: the number of reports containing the suspect drug with other adverse events (except the event of interest); c: the number of reports containing the suspect adverse events with other medications (except the drug of interest); d: the number of reports containing other medications and other adverse events; N: the number of all reports. N: the number of all reports.
