# Supplementary material for: Pharmacovigilance profiles of three generations of mineralocorticoid receptor antagonists and network toxicology analysis
Source: Front Med (Lausanne). 2026 Jun 23;13:1797331. doi: 10.3389/fmed.2026.1797331 (PMC13337816; doi:10.3389/fmed.2026.1797331)
Supplement: Supplementary file 8 [file Data_Sheet_2.docx]

**Supplementary Table 2.** Formulas and thresholds of the four algorithms.

| **Algorithms** | **Equation** | **Criteria** |
| --- | --- | --- |
| ROR | 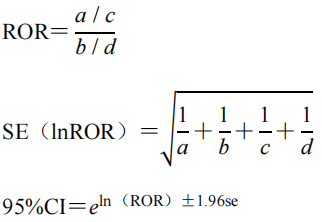 | a ≥ 3，ROR ≥ 3，95%CI（low limit）＞ 1 |
| PRR | 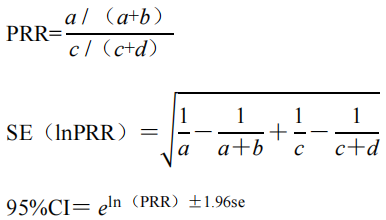 | A ≥ 3，PRR ≥ 2，95%CI（low limit）＞ 1 |
| BCPNN | 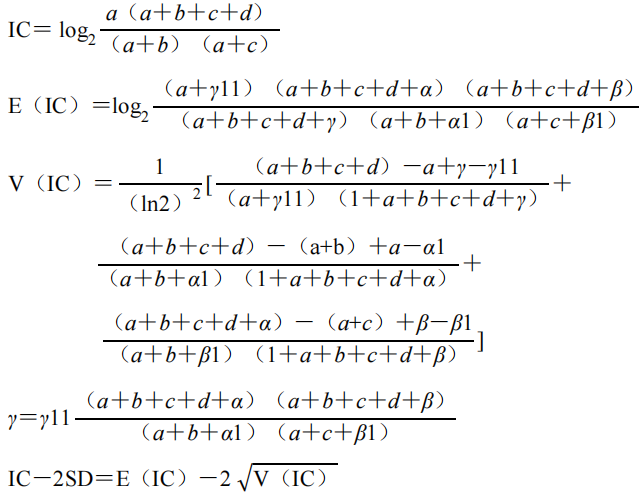 | IC025 ＞ 0 |
| EBGM | 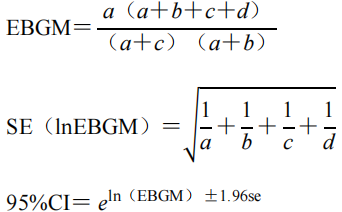 | EBGM05 ＞ 2 |

Note: a: the number of reports containing both the suspect drug and the suspect adverse events; b: the number of reports containing the suspect drug with other adverse events (except the event of interest); c: the number of reports containing the suspect adverse events with other medications (except the drug of interest); d: the number of reports containing other medications and other adverse events; N: the number of all reports. Abbreviations: ROR, Reporting Odds Ratio; PRR, Proportional Reporting Ratio; BCPNN, Bayesian Confidence Propagation Neural Network; EBGM, Empirical Bayes Geometric Mean.
