# Supplementary material for: Pharmacovigilance profiles of three generations of mineralocorticoid receptor antagonists and network toxicology analysis
Source: Front Med (Lausanne). 2026 Jun 23;13:1797331. doi: 10.3389/fmed.2026.1797331 (PMC13337816; doi:10.3389/fmed.2026.1797331)
Supplement: Supplementary file 9 [file Data_Sheet_3.docx]

**Supplementary Table 3.** The criteria and corresponding scores used to prioritize ADRs identified through disproportionality analysis.

| **Criterium** | **2 Points** | **1 Point** | **0 Points** |
| --- | --- | --- | --- |
| Reporting rate (cases/non-cases) | >10% | 1–10% | 0-1% |
| Signal stability (consistency across disproportionality analyses) | ≥3of 4 | 2 of 4 | ≤1of 4 |
| Reported case fatality rate (proportion of reports with death as outcome) | >50% | 25–50% | <25% |
| Clinical relevance (serious likely drug-attributable ADRs) | DME | IME | None |

Abbreviations: ADRs, adverse drug reactions; DME, designated medical event; IME, important medical event.
