# Supplementary material for: Pharmacovigilance profiles of three generations of mineralocorticoid receptor antagonists and network toxicology analysis
Source: Front Med (Lausanne). 2026 Jun 23;13:1797331. doi: 10.3389/fmed.2026.1797331 (PMC13337816; doi:10.3389/fmed.2026.1797331)
Supplement: Supplementary file 10 [file Data_Sheet_4.DOCX]

**Supplementary Table 4.** Significant signal strength of ADRs at the SOC level.

| **Items** | **SOC** | **Cases** | **ROR(95%Cl)** | **PRR(X^2^)** | **EBGM**  **(EBGM05)** | **IC**  **(IC025)** |
| --- | --- | --- | --- | --- | --- | --- |
| spironolactone | General disorders and administration site conditions | 3255 | 0.7 ( 0.68 - 0.73 ) | 0.74 ( 358.19 ) | 0.74 ( 0.71 ) | -0.43 ( -0.49 ) |
|  | Metabolism and nutrition disorders | 2979 | 6.03 ( 5.8 - 6.27 ) | 5.44 ( 10999.5 ) | 5.43 ( 5.22 ) | 2.44 ( 2.38 ) |
|  | Investigations | 1895 | 1.25 ( 1.19 - 1.31 ) | 1.23 ( 87.68 ) | 1.23 ( 1.17 ) | 0.3 ( 0.23 ) |
|  | Renal and urinary disorders | 1883 | 4.23 ( 4.04 - 4.44 ) | 3.99 ( 4295.09 ) | 3.99 ( 3.8 ) | 2 ( 1.92 ) |
|  | Gastrointestinal disorders | 1796 | 0.82 ( 0.78 - 0.86 ) | 0.84 ( 63.28 ) | 0.84 ( 0.8 ) | -0.26 ( -0.33 ) |
|  | Nervous system disorders | 1786 | 0.83 ( 0.79 - 0.87 ) | 0.84 ( 58.9 ) | 0.84 ( 0.8 ) | -0.25 ( -0.32 ) |
|  | Injury, poisoning and procedural complications | 1531 | 0.55 ( 0.52 - 0.57 ) | 0.57 ( 544.13 ) | 0.57 ( 0.54 ) | -0.8 ( -0.88 ) |
|  | Skin and subcutaneous tissue disorders | 1447 | 1.05 ( 1 - 1.11 ) | 1.05 ( 3.7 ) | 1.05 ( 1 ) | 0.07 ( -0.01 ) |
|  | Cardiac disorders | 1435 | 2.25 ( 2.14 - 2.38 ) | 2.18 ( 943.77 ) | 2.18 ( 2.07 ) | 1.13 ( 1.05 ) |
|  | Respiratory, thoracic and mediastinal disorders | 931 | 0.78 ( 0.73 - 0.83 ) | 0.78 ( 58.54 ) | 0.78 ( 0.73 ) | -0.35 ( -0.45 ) |
|  | Psychiatric disorders | 897 | 0.62 ( 0.58 - 0.66 ) | 0.63 ( 203 ) | 0.63 ( 0.59 ) | -0.66 ( -0.76 ) |
|  | Vascular disorders | 854 | 1.62 ( 1.51 - 1.73 ) | 1.6 ( 195.16 ) | 1.6 ( 1.49 ) | 0.68 ( 0.57 ) |
|  | Reproductive system and breast disorders | 845 | 3.93 ( 3.67 - 4.21 ) | 3.83 ( 1778.97 ) | 3.82 ( 3.57 ) | 1.94 ( 1.83 ) |
|  | Musculoskeletal and connective tissue disorders | 715 | 0.53 ( 0.49 - 0.57 ) | 0.55 ( 284.96 ) | 0.55 ( 0.51 ) | -0.87 ( -0.98 ) |
|  | Infections and infestations | 504 | 0.37 ( 0.34 - 0.4 ) | 0.38 ( 537.39 ) | 0.38 ( 0.35 ) | -1.39 ( -1.52 ) |
|  | Blood and lymphatic system disorders | 412 | 0.97 ( 0.88 - 1.07 ) | 0.97 ( 0.38 ) | 0.97 ( 0.88 ) | -0.04 ( -0.19 ) |
|  | Immune system disorders | 361 | 1.3 ( 1.17 - 1.44 ) | 1.29 ( 24.28 ) | 1.29 ( 1.17 ) | 0.37 ( 0.22 ) |
|  | Hepatobiliary disorders | 302 | 1.3 ( 1.16 - 1.46 ) | 1.3 ( 20.96 ) | 1.3 ( 1.16 ) | 0.38 ( 0.21 ) |
|  | Product issues | 281 | 0.67 ( 0.6 - 0.75 ) | 0.67 ( 45.12 ) | 0.67 ( 0.6 ) | -0.57 ( -0.74 ) |
|  | Eye disorders | 251 | 0.49 ( 0.44 - 0.56 ) | 0.5 ( 128.5 ) | 0.5 ( 0.44 ) | -1 ( -1.18 ) |
|  | Neoplasms benign, malignant and unspecified (incl cysts and polyps) | 233 | 0.35 ( 0.31 - 0.4 ) | 0.36 ( 274.73 ) | 0.36 ( 0.32 ) | -1.48 ( -1.67 ) |
|  | Congenital, familial and genetic disorders | 153 | 2.02 ( 1.72 - 2.37 ) | 2.02 ( 78.46 ) | 2.01 ( 1.72 ) | 1.01 ( 0.77 ) |
|  | Ear and labyrinth disorders | 130 | 1.19 ( 1 - 1.41 ) | 1.19 ( 3.87 ) | 1.19 ( 1 ) | 0.25 ( -0.01 ) |
|  | Endocrine disorders | 129 | 1.99 ( 1.67 - 2.37 ) | 1.98 ( 63.07 ) | 1.98 ( 1.67 ) | 0.99 ( 0.72 ) |
|  | Surgical and medical procedures | 121 | 0.35 ( 0.29 - 0.41 ) | 0.35 ( 147.91 ) | 0.35 ( 0.29 ) | -1.51 ( -1.77 ) |
|  | Pregnancy, puerperium and perinatal conditions | 79 | 0.74 ( 0.6 - 0.93 ) | 0.75 ( 6.87 ) | 0.75 ( 0.6 ) | -0.42 ( -0.74 ) |
|  | Social circumstances | 54 | 0.45 ( 0.34 - 0.59 ) | 0.45 ( 36.34 ) | 0.45 ( 0.35 ) | -1.15 ( -1.52 ) |
| eplerenone | General disorders and administration site conditions | 217 | 0.73 ( 0.63 - 0.84 ) | 0.76 ( 19.03 ) | 0.76 ( 0.66 ) | -0.39 ( -0.59 ) |
|  | Investigations | 166 | 1.75 ( 1.49 - 2.05 ) | 1.67 ( 47.55 ) | 1.67 ( 1.42 ) | 0.74 ( 0.5 ) |
|  | Nervous system disorders | 144 | 1.05 ( 0.89 - 1.25 ) | 1.05 ( 0.37 ) | 1.05 ( 0.88 ) | 0.07 ( -0.18 ) |
|  | Gastrointestinal disorders | 114 | 0.81 ( 0.67 - 0.98 ) | 0.82 ( 4.82 ) | 0.82 ( 0.68 ) | -0.28 ( -0.56 ) |
|  | Renal and urinary disorders | 108 | 3.72 ( 3.06 - 4.52 ) | 3.54 ( 200.68 ) | 3.54 ( 2.91 ) | 1.82 ( 1.5 ) |
|  | Injury, poisoning and procedural complications | 104 | 0.58 ( 0.47 - 0.7 ) | 0.6 ( 30.38 ) | 0.6 ( 0.49 ) | -0.73 ( -1.02 ) |
|  | Cardiac disorders | 103 | 2.52 ( 2.07 - 3.08 ) | 2.43 ( 88.6 ) | 2.43 ( 1.99 ) | 1.28 ( 0.97 ) |
|  | Metabolism and nutrition disorders | 103 | 3.03 ( 2.49 - 3.7 ) | 2.91 ( 131.58 ) | 2.91 ( 2.38 ) | 1.54 ( 1.22 ) |
|  | Skin and subcutaneous tissue disorders | 78 | 0.87 ( 0.69 - 1.09 ) | 0.88 ( 1.43 ) | 0.88 ( 0.7 ) | -0.19 ( -0.52 ) |
|  | Respiratory, thoracic and mediastinal disorders | 73 | 0.95 ( 0.75 - 1.2 ) | 0.95 ( 0.19 ) | 0.95 ( 0.75 ) | -0.07 ( -0.41 ) |
|  | Vascular disorders | 71 | 2.11 ( 1.66 - 2.67 ) | 2.06 ( 39.4 ) | 2.06 ( 1.62 ) | 1.04 ( 0.67 ) |
|  | Psychiatric disorders | 69 | 0.74 ( 0.58 - 0.95 ) | 0.75 ( 5.89 ) | 0.75 ( 0.59 ) | -0.41 ( -0.76 ) |
|  | Musculoskeletal and connective tissue disorders | 52 | 0.6 ( 0.46 - 0.79 ) | 0.61 ( 13.23 ) | 0.61 ( 0.47 ) | -0.7 ( -1.09 ) |
|  | Infections and infestations | 42 | 0.48 ( 0.35 - 0.65 ) | 0.49 ( 23.42 ) | 0.49 ( 0.36 ) | -1.03 ( -1.46 ) |
|  | Blood and lymphatic system disorders | 33 | 1.21 ( 0.86 - 1.7 ) | 1.2 ( 1.16 ) | 1.2 ( 0.85 ) | 0.27 ( -0.24 ) |
|  | Reproductive system and breast disorders | 30 | 2.12 ( 1.48 - 3.05 ) | 2.1 ( 17.5 ) | 2.1 ( 1.47 ) | 1.07 ( 0.5 ) |
|  | Eye disorders | 24 | 0.74 ( 0.49 - 1.1 ) | 0.74 ( 2.24 ) | 0.74 ( 0.49 ) | -0.44 ( -1 ) |
|  | Neoplasms benign, malignant and unspecified (incl cysts and polyps) | 21 | 0.49 ( 0.32 - 0.76 ) | 0.5 ( 10.76 ) | 0.5 ( 0.33 ) | -1 ( -1.59 ) |
|  | Ear and labyrinth disorders | 19 | 2.71 ( 1.72 - 4.26 ) | 2.69 ( 20.23 ) | 2.69 ( 1.71 ) | 1.43 ( 0.66 ) |
|  | Hepatobiliary disorders | 16 | 1.07 ( 0.65 - 1.74 ) | 1.07 ( 0.07 ) | 1.07 ( 0.65 ) | 0.09 ( -0.62 ) |
|  | Surgical and medical procedures | 12 | 0.53 ( 0.3 - 0.94 ) | 0.54 ( 4.83 ) | 0.54 ( 0.3 ) | -0.89 ( -1.65 ) |
|  | Endocrine disorders | 11 | 2.63 ( 1.45 - 4.76 ) | 2.62 ( 11.04 ) | 2.62 ( 1.45 ) | 1.39 ( 0.37 ) |
|  | Immune system disorders | 7 | 0.39 ( 0.18 - 0.81 ) | 0.39 ( 6.82 ) | 0.39 ( 0.18 ) | -1.36 ( -2.27 ) |
|  | Product issues | 5 | 0.18 ( 0.08 - 0.44 ) | 0.19 ( 18.15 ) | 0.19 ( 0.08 ) | -2.43 ( -3.4 ) |
|  | Social circumstances | 4 | 0.52 ( 0.19 - 1.38 ) | 0.52 ( 1.81 ) | 0.52 ( 0.19 ) | -0.95 ( -2.1 ) |
|  | Congenital, familial and genetic disorders | 3 | 0.61 ( 0.2 - 1.9 ) | 0.61 ( 0.74 ) | 0.61 ( 0.2 ) | -0.71 ( -2.01 ) |
|  | Pregnancy, puerperium and perinatal conditions | 2 | 0.29 ( 0.07 - 1.17 ) | 0.29 ( 3.44 ) | 0.29 ( 0.07 ) | -1.77 ( -3.05 ) |
| finerenone | Investigations | 770 | 5.84 ( 5.38 - 6.35 ) | 4.51 ( 2241.23 ) | 4.51 ( 4.15 ) | 2.17 ( 2.05 ) |
|  | Metabolism and Nutrition Disorders | 268 | 4.76 ( 4.2 - 5.4 ) | 4.4 ( 720.31 ) | 4.4 ( 3.88 ) | 2.14 ( 1.94 ) |
|  | Renal and Urinary Disorders | 277 | 5.76 ( 5.09 - 6.52 ) | 5.29 ( 981.63 ) | 5.29 ( 4.67 ) | 2.4 ( 2.2 ) |

Abbreviations: ROR, Reporting Odds Ratio; PRR, Proportional Reporting Ratio; CI, confidence interval; EBGM, Empirical Bayes Geometric Mean; IC, Information Component; SOC, system organ classes.
